# Supplementary material for: A Potentially Practicable Halotolerant Yeast Meyerozyma guilliermondii A4 for Decolorizing and Detoxifying Azo Dyes and Its Possible Halotolerance Mechanisms
Source: J Fungi (Basel). 2023 Aug 15;9(8):851. doi: 10.3390/jof9080851 (PMC10456123; doi:10.3390/jof9080851)
Supplement: Supplementary file 1 [file jof-09-00851-s001.zip › jof-2535121-supplementary.pdf]

Supporting Information for:

A potentially practicable halotolerant yeast *Meyerozyma guilliermondii* A4 for decolorizing and detoxifying azo dyes and its possible halotolerance mechanisms

Yue Feng 1,†, Jingru Cui 1,†, Bingwen Xu 2, Yifan Jiang 1, Chunqing Fu 1 and Liang Tan 1,*

1 Key Laboratory of Plant Biotechnology of Liaoning Province, School of Life Science, Liaoning Normal University, Dalian 116081, China; fss990214@163.com (Y.F.); acui0315@163.com (J.C.); jiangyifan202203@163.com (Y.J.); 15560356752@163.com (C.F.)

2 Dalian Center for Certification and Food and Drug Control, Dalian 116037, China; xubingwen0402@126.com

* Correspondence: tanliang19811210@163.com or tanliang1210@lnnu.edu.cn; Tel./Fax: +86-411-85827068

† These authors contributed equally to this work.

This 28-page file includes:

3 Supplemental Tables

5 Supplemental Figures

2 Supplemental Texts

4 References

Table S1 Azo dyes used in the present study and their main chemical information.

Name (CAS number)	Chemical structure	Molecular weight	Characteristic absorption wavelength (nm)
Acid Red B (3567-69-9)	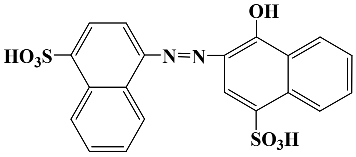	458.5	516
Acid Orange II (633-96-5)	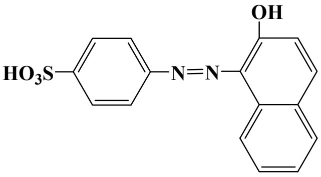	328.3	484
Acid Scarlet GR (5413-75-2)	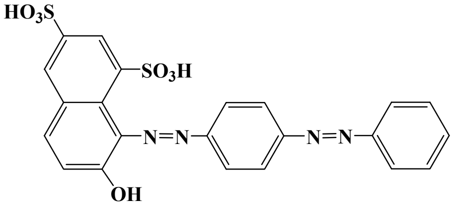	512.5	511
Acid Red 3R (1082572-30-2)	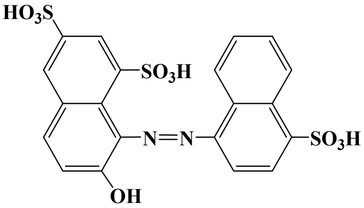	538.5	507
Reactive Brilliant Red K-2G (12238-01-6)	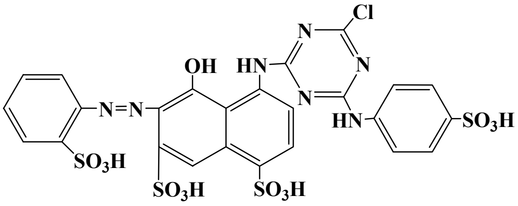	788.2	509
Reactive Violet KN-4R (12226-38-9)	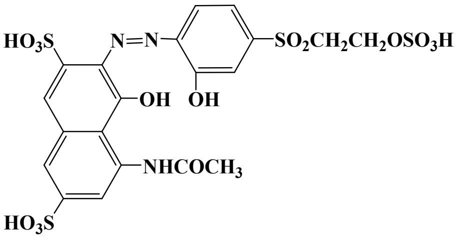	669.6	558
Reactive Yellow 3R (93050-80-7)	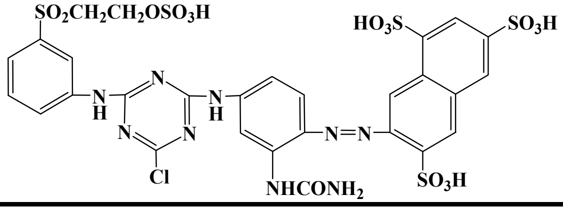	938.3	540

Table S2 Sequences of the target genes which were validated through QRT-PCR.

Gene ID	Genetic sequences
D_102776	ATGGCAGAATTTTATAGACACCTTCTTTCCCAGCTCTTATTGTTGGTGCCTGCCTCCAAGTTCCAGCCGCCCTCTTCAGATGACCCAGACCCTCA<br>CACAGAGGTTATTGACGTAGTTAGTGCAATTCTCGCTGGCAACTCGGGTTCCATTTTCACAGATACTAGGTATAAAGGAGTGTTTTACAATGCTA<br>ATGCTTTACGTGTGTTATCCCAGTATCCCAATATAAGCTTGAAGAGTGCATTACACGGCTATGTGGACGAAATTATCAAGTCTGTGGAACAGAGC<br>TCTTCGACCGATTCTCGACTTTTAGTGCAATTAACTGCAATTGTTGCATTGCAATTATTCATCCAGCTCAATTTTACTGGACCAAATGTTGACTTTA<br>AGGCTCACGAGCTCTTAATGCCCCATGCTGACTACGAAAATTTGCACTTGGACCTCATAGACTTGCTCACCGTTGAGGGCCAACAGGCATATGA<br>GCTTATGAATGAACCATTGCTTTTCATTATAGCCAGTTTGATGTTTGAAAGATTGATGAATGTGGGACCACAGTACTCGTTTATTGGCAAAGATAT<br>GAACGTAGAGCTTGACCAAATGGTAGAAGCCACTAGACCTTTGGTCGCCAATCCAAGCATAGAAGACGCTTCGCTTTTCTGGTGGAGATCACG<br>AGCGTTGCAAATCCACATCTCGCTCCTATCAGAACCTCCAAGTGTTTTGACGAGTATATCGGCACTTTTGCTCGGTCCTTCCACGGCAGAAGTTT<br>TGGCTCATGGAAATGACACTCTTCTGTCACTTGTCAAATACGAGTTTCTCATAGAAACCGCTCGAGCCAGCATTCATTCATACACAGAACATTTG<br>GCTACCTCACTTTTGGCGAGAGCATCAGAGGTTACTGAGTTGCAATTTGTGTTGACTGGAGCTAAGGCTAAACGAACAAAGTTCCAAACATTCA<br>CCACATCTTCGTTGGTATTGCTAGCAAAGAGCAAAGGGTCTACTTTACTCGATGAAGACACGGCCGAGGAACCAGCAAAGTTCAATTTGGATTC<br>AGACCTTCTTCTCGAACGTCCACAGTACGATGCTCTCGAAGACATAAAAGAACCCGTTTCTAAAAAGACCAAATTTGAAGATGAGGACCAGAA<br>AAACGAAGTTCCGCTCTTGCCTATAGCACGTCACCAAGACCAAATTCCTTTGGAGCTTTCAGCCTTGGATCCCAACGATCAACCCGCATTGAAC<br>GACTTGGACAACCTTCAGCTTTTGCTCCGTTTGATGGTGCTTCGCCAATCTTCGCCAGCTAGCAATTCGCTTGTCGAAGAAGAGCTCATGGCAA<br>TTGTGAGCAGAGTCATATATCAACCATCGAAATCTGTCAACTGGGCCATTTTTGGAAGGGCGTTATGGGAGAGATCACTATTGGAAACAAGCAA<br>GGCTCGGACCGTGGAAAGAGGAATATTACAGATGACATCGTTAGTTGAAGAAATCGGTCTCAAGATTAAAACCAGGCTTTTGCCTCAGGAAATG<br>GAAGCGAACAACTCTTCCCCAGCTGCTTCTCGGCTCCGGTTCATCCACCAACTTCCGTTGATGCCACAGTGGACCATGGACGTTAAGTTGGCAG<br>AAAAGTACATGTCTTTGGGAGTGTTGAAGTCTGCGTTGGAAATCTACGAAAGACTTCATTTGGAATGCGAAGTGGCACTTTGTCATGCTGCGGT<br>TGACAACGAAACTGAGGCGGAAAACGTCCTCTTACGTCGTTTAGAACAGCACCCCAACGATGCCAGAGCCACATCAATTCTCGGTGATATTCG<br>CCAGGACCCTCAGCTCTGGGAACGAGCCTGGGAAATTGGAAGGTACTCAAAAGCAAAGAACTCGTTATCGAGGTATTACTATAACCCTCCAGC<br>AAATTCTGGACTTTCCAAAGACTTGGAAGCAGCACTTTTACATATGAGTGATTGTTTGAGGGCCGACCCACTTAATTTTGAAAATTGGTTCTTTT<br>ACGGCTGTTGTGGACTCGAAACTGCAAACTACGAATTAGCATCGGAGGCATTTACTCGTTGTGTTTCGTTAGATGACTCGAATTCGCATGCTTGG<br>TCCAACTTGGCTACGGCGCTTCTAAGATTAGACAAGACCAGACCAGCATTCAATGCTTTGAAAAAGGCAATGGTGAGTTCAAAAGAAGGTAAG<br>CGGTCTTGGAGAATTCATGAAAACTTTGTCATTGTTGCCATGAAACTCAATGAATGGTCTGATGTCTTACAAGCGACCAGAGAATTGATAGACAT<br>GAAAGAAGGTGGAGAGTCAAGTATCGATATTCCTGTCATTGAAAAGTTGGTGGAAATTCTCGTGGCCACAGATTATCCTAAGGAGGGAGAGAG<br>ATTGACTCATTATCAGAACTCGTGTATTGATTTGGTGTGCAATATGCTTCCTAATGTAATTACCAACTCTGGTAGATGTTGGAGAATTGTTGCTCGA<br>GTTCATATCTGGAGAAAGAAGCCATGGGAGGCATTGGAATGCTACGAGAAAGCTTATCGAGCGGTATCCCAAAAGCATGATTTGAGCACCAACG<br>AAACCATTTGGAATGAAGCTGTAGAGGCTTGTGAAGACTTGGTCGCAGCATATGAGTCTCTTGGAGAGCTTCCAGGCAAACATGGAGCCGACG<br>ATTTAGTGTGCAAGGACTGGAAATACAAAAGCAAAACGACAATCAGATCGTTAATGTCGAAGGGAAAGCTGATGTGGGAAGATAGTCAAGGCT<br>GGGATCGCTTAATGGCGCTCAAAGAAAACTTATGA
D_105169	ATGTCGTACGATAATAATCACAACTATTATGACCCGAATCAGCCGATGGGTAATGATGGTTATTATCAACAACAACCTTATGATGATATGAACCAA<br>CAACCACATCAGGATTACTATGACCCCAACATGCAATATCAACAACAGCCTTATGACATGGACGGGTACCAAGATCCCAACTATCAGGGTCAAC<br>AGATTCCTCAACAAGGATATAACGCTGATCCTGAAGCGTTTTCTGATTTCAGTTACGGGGGTCAAACTCCAGGTACTCCTGGTTACGACCAATAC

GGTACTCAGTTTACCCCTTCTCAGATGAGTTATGGTGGTCCCAGATCGTCTGGTGCTTCTACACCCATCTACGGTGGAGCAGGTGGAAACTACGA
TCCAACCCAATTTCAAATTTCCTCCAGCATGCCTTACCCTGCTTGGTCTGCCGATGCGCAAGCACCAGTTAAAGTAGAGCATATTGAGGATATTT
TCATCGATTTGGCCAACAAGTTTGGTTTCCAAAGAGATTCGATGAGAAATATGTTCGACTACTTTATGACATTATTAGACTCTAGATCCTCGCGTA
TGTCTCCCGCCCAAGCCTTATTGAGTTTACACGCGGATTACATTGGAGGTGAAAACGCCAACTACAAAAAGTGGTATTTTGCTTCGCAACAGGA
TTTGGATGAATCAATTGGTTTTGCCAACATGAAAATGGGAAAGGTTGGTAAGAAAGCTAGAAAGGCATCCAAGAAGTCCAAGAAGGCAAGAA
AGGCAGTCGAGGAACATGGCCAGGACATCGACGCTTTGGCTAATGAATTAGAAGGCGACTACTCTTTGGAAGCCGCAGAAATTAGATGGAAAG
CCAAGATGAACTCTTTGTCTCCTGAAGAGAGAATTAGAGACATTGCTTTGTATTTGTTGTTGTGGGGTGAAGCCAATCAAGTTAGATTTACTCCT
GAAGCCATTTGTTACATCTACAAAACGGCTTTCGATTACTTGGTGTCTCCTCAATGTCAACAAAGACAAGAGCCTGTTCCAGAAGGTGATTACTT
GAATCGTGTCATCACACCTTTGTACCGATTCTTTAGATCTCAAGTCTACGAAATCTACGAGGGCCGCTTGGTCAAGCGTGAAAAGGATCACAATA
AGGTGATTGGTTACGATGATGTTAACCAGTTGTTCTGGTACCCAGAAGGTATTTCAAGAATTATCTTTGAAGATGGTACTAGATTGATTGACGTCC
CACCTGAAGAGCGTTACTTGAGATTTGGTGAGGTCGAATGGCACAATGTCTTCTTCAAGACTTACAAGGAAATTAGAACTTGGTTGCATCTTATC
ACCAACTTCAACAGAATCTGGATTATCCACGGTACCGTCTACTGGATGTATACTGCTTACAACTCCCCAACGTTGTACACTCTCCATTATGTTCAA
ACTATTAACAATCAGCCTCTTGCTTCATCAAGATGGGCTGCCGCTGCTATTGGCGGTGTTTTGGCTGCCTTCATTCAAATTGCTGCAACGGTGTG
TGAGTGGATGTTCGTTCCTAGAGAGTGGGCTGGTGCTCAACATTTGACACGTCGTTTGATGTTCCTCATTATAATTTTCGTGGTTAACTTGGCCCC
AGTTGTTTTCACCTTTTACTACGCTGGCTTGGCTGCAAAGTCAAAAGCAGCGCTAGTGGTTTCGATTGTTGGGTTCTTCATTGGTATTGCTACGA
TCGTATTCTTTGCCGTTATGCCTTTGGGTGGTTTGTTCACAAGTTATATGAACAGAAGATCAAGAAGATATTTGGCTTCTCAAACCTTTACTGCCA
ACTTCAACAAGTTGACTGGCTTAGACATGTGGCTCTCGTATTTATTGTGGGTTCTTGTGTTCTTTGCCAAGTACATCGAATCTTATTTCTTCATGG
CTTTGTCCTTGAGAGATCCCATTAGAACCTTGTCTACCACTAACATGAGATGTATCGGTGAAGTCTGGTTCGGCGACAAATTGTGTAAACACCAA
GCTAAGGTCGTCTTGGGTTTGATGTACCTTGTGGACTTGTTATTGTTCTTTTTGGATACCTACATGTGGTACATTATCTGTAACTGTGTGTTTTCCA
TTGGTCGTTCATTCTACTTGGGTATTTCTATCTTAACACCTTGGAGAAACATTTTCACAAGACTTCCAAAGAGAATCTACTCTAAGATTTTGGCTA
CCACTGAGATGGAGATTAAGTACAAACCAAAGGTTTTGATCTCTCAAATCTGGAATGCTATTGTGATTTCCATGTACAGAGAACATTTGTTGGCT
ATTGATCACGTTCAAAAGTTGTTGTACCACCAAGTTCCATCTGAAATTGAGGGTAAGAGAACTTTGAGAGCTCCAACTTTCTTCGTTTCTCAGG
ATGACAATAACTTCGAGACGGAGTTCTTCCCAAGAAACTCTGAAGCTGAGAGACGTATCTCTTTCTTTGCTCAGTCCTTGGCTACACCAATCTT
GGAACCATTGCCAGTTGACAACATGCCTACCTTTACTGTGTTTACTCCTCACTACTCTGAAAGAATTTTATTGTCTTTAAGAGAAATTATTAGAGA
GGATGACCAATTCTCCCGTGTTACCTTGTTAGAATATTTGAAACAATTGCACCCAGTTGAATGGGACTGTTTTGTCAAGGACACCAAAATTTTGG
CGGAAGAAACTGCTGCTTTCGAAGGTGAAGGTGTCGACAAAGAGTCTGAAGATGGCCTCAAATCTAAGATTGATGATTTGCCATTCTACTGTAT
TGGTTTCAAGTCTGCTGCTCCTGAGTATACATTGAGAACCCGTATTTGGGCTTCCTTGAGATCCCAAACCTTGTACCGTACCGTTTCCGGTTTCAT
GAACTATGCCAGAGCCATCAAATTGTTGTACCGTGTTGAGAACCCAGAATTGGTTCAATATTTCGGTGGAGACCCAGAGGGCCTTGAACTCGCA
TTGGAGAGAATGGCAAGAAGAAAATTCAAGTTTGTTGTTTCAATGCAAAGATTGGCCAAGTTTGAAGACTGGGAGATGGAGAATGCTGAATTT
TTGTTACGTGCTTACCCTGACTTGCAAATCGCTTACTTAGACGAAGAGCCTGCCTTATCCGAAGAGGAAGATCCAAGAATCTATTCCGCTTTGAT
TGATGGTCACTGTGAAATTTTGGAGAATGGTAGACGTCGTCCTAAGTTTAGAGTGCAATTGTCTGGTAACCCAATTTTGGGTGATGGTAAGTCTG
ATAACCAAAATCATGCTTTGATTTTCCACCGTGGTGAGTACATTCAATTGATTGATGCCAATCAAGACAACTACTTGGAAGAATGTTTGAAGATC
AGATCTGTGTTGGCCGAATTCGAAGAATTGAACGTTGAACAAGTTAACCCATATGCTCCTAACTTGAAGACTGATGTCAAGACTGGAAACAATG
CCCCTGTTGCTATTTTGGGTGCTCGTGAATACATTTTCTCAGAGAACTCTGGTGTTTTGGGTGATGTCGCTGCTGGTAAGGAACAAACTTTCGGT
ACCCTTTTCGCAAGAACTTTGGCACAGATTGGTGGTAAATTGCATTATGGACATCCTGATTTCTTGAATGCCACTTATATGTTCACCAGAGGAGG
TGTTTCTAAGGGACAAAAAGGATTGCATTTGAACGAAGATATTTATGCTGGTATGACTGCTATGTTAAGAGGTGGTAGAATCAAGCACTGTGAAT
ACTACCAGTGTGGTAAGGGTAGAGATATGGGTTTCGGATCCATTTTGAATTTCACAACCAAGATTGGTGCTGGTATGGGAGAACAGATGTTGTCT
CGTGAATATTACTACTTGTCGACTCAGCTTCCTTTGGACAGATTTTTGTCCTTCTACTACGGTCATCCAGGTTTCCATATTAACAACTTGTTCATTC

AATTATCTTTGCAAGTTTTCATGTTGGTGTTGGCTAACTTGAACTCATTGGCTCACGAATCCATTTTGTGCTCCTACAACAGAAACACTCCTATCA
CCGATGTGTTATATCCTTACGGTTGTTACAACTTCGCTCCCGCAGTTGACTGGATTAGACGTTACACATTGTCTATTTTCATTGTGTTCTTCATTGC
GTTTATTCCATTGGTGGTTCAAGAATTGATTGAAAGAGGTGTCTTGAAGGCAGCCCAAAGATTCTGTCGCCATCTTATCTCGTTGTCGCCAATGT
TCGAAGTGTTCGTTGCTCAAATCTACTCGACTTCATTGATTACTGATTTGACTGTTGGTGGTGCCAGATATATCTCCACTGGTAGAGGTTTCGCCA
CTTCTCGTATTCCATTCTCCATTTTGTACTCCCGTTTTGCTGATTCATCGATTTACATGGGTTCAAGATCTATGTTGATTTTGTTGTTCGGAACAGTT
TCTCACTGGCAAGCTCCATTGTTGTGGTTCTGGGCATCGTTGTCATCATTGATGTTCTCACCATTTATTTTCAATCCTCATCAATTCTCTTGGGAAG
ATTTCTTCATCGATTACCGTGACTTTATCAGATGGTTATCCCGTGGTAACTCCAAGTGGCATAGAAACTCTTGGATTGGTTACGCTCGTCTTTCGA
GATCTCGTGTCACTGGTTTCAAGAGAAAGTTGACTGGCGATGTATCGGAGAAGGGTGCTGGTGACGCTTCCAGAGCACACAGATCCAACATTT
TCTTTGCTGACTTTTTGCCTACTTTGATCTACACTGCTGGTCTTTTTGTTGCCTACACTTTTATCAACGCCCAGACTGGTGTAAGAAGGGGACCA
AATGGTGAGGGAAACCCTACTGAGGTCAACTCTGTGTTGAGAGTTGTGATTTGTTCTTTGGCTCCTGTAGTTATTGATCTCGGTGTTTTGGCAGG
ATGTCTTGGTCTCGCTTGTTGCGCAGGTCCATTATTGGGATTGTGCTGCAAGAAGACTGGTTCTGTGATTGCCGGTGTTGCTCATGGTATTGCCG
TTGTTGTGCACTTGGTCTTTTTCATTGTCATGTGGGTCTTGGAAGGCTTCAACTTTGCTAGAATGTTATTGGGTCTTTGTACCATGGTTTACATCC
AAAGATTGTTGTTCAAGTTTTTGACGATTTGCTTCTTGACCAGAGAGTTCAAGAACGATAAATCTAACCAAGCTTTCTGGACCGGTAAATGGTA
CGGATCTGGTTTGGGATGGATGGCGTTCACCCAACCAGCTCGTGAATTCTGTGCCAAGGTTATTGAAATGTCGGAGTTTGCCGGAGACTTTATTT
TGGCACACATCATCTTGTTTGTGCAATTGCCTATCATCTGTATTCCATTGATTGACAGATGGCATTCGACCATGTTGTTCTGGTTGAAACCTTCCA
GGTTGATCAGACCTCCAATCTACTCATTGAAGCAGGCCAAGTTGAGAAAGAGAATGGTGAGAAGATACTCTACTCTTTACTTTTTGATTTTGGTT
ATCTTTGTTGCACTTATTGCTGCACCTGCTGCTATTGGTAACACCACTCTTGTACCAGAAGACTTGGGTAAATCGTTGAAGGGTACTTTTGCTGG
ATTGTTCCAACCCAGACACGTTGAAAACAATGACACTGGTACTTCGGTGGGCAAGGCTTACATGAGTCTGCACACATTCTTGACCAAAATGTTC
AGTGGGACGACGACCAAATATTCCACCAAGCCATAA

D_105067 ATGTCCCAACTAAACTCTCGCGCTAGCAGGTCTTCTTTTTACGTTGCTTTGACTACAATACATCGAGACTACGTTGGAACCTCTTCAAACTACAG
GAAATGGCTCAAGGCAGCTTGCAAACAAGATGGGAGTGATGGGCCCGAAAGGATAATTAAAAACGAAAACATTAATACAGCTTGCAAGATGTA
CGTAACTGAAGTTGCCTTGTACCTCTTAATATGGGGAGAAGCCAGCAATATCAGATTCATGCCAGAATGCATTTGCTTCATTTACAAGTGCTGTCT
CGACTACTATATGGCAGAGGACAGGATCACGATTGCAAAACCATTTCTTGACCACACGATTGTACCTTTGTTTGAATTCTTGAGGGAACAACAAT
ACAAACTAAAAGACGGAAACTGGATTCGGAGAAGGCGGGACCATGCTCGTATCATAGGATACGACGATATGAATCTGTTTTTCTGGTATAATGA
AAACCTCCAGAAATTAGTGGTCGATTCGGGCAGGCTCTATGATATGGCTGCACTGGACCGATACCCAAGCTTTGACAAAATTGATTGGAACAAA
GCTTTCTTCAAATCCTACAGGGAAGTACGCACCTGGTCTCACCTTTTGACCAACTTCAGTAGGGTATGGATTACACACTTGACAATGTTCTGGTA
CTTTACCAGTTGCAATTCACTCTCATTGTACACAAAAGAATACAGCCCAGAGTACGACAACACGCCTCCACCTCATGTTATTTGGTCAGTGGTTT
CATTAGGTGGAGTATTAGCATCGACAATTGCCCTTGTGAGTTGCTTGATGGAACTTAGATTCGTACCAAGAAAGTTTCCAGGAGCGCCATCTGTA
TTGGGAAGAAGTTTACTTCTCATGGCTCTTCTGGCCTTGAATCTTGGACCGTCCTTTTATCTTTTGTGGATATTGCCTGCTGATGTCTATTCAAGGT
CGGGTCACCTAATAGGGATCATTCAGTTTGGGATTTCTGCTGCCACTTTCCTATACCTAGTGCTTGTTCCTCCCGCACAATACTTCTCTTGCATACT
TCCATCCCAGCCCAATTCACATCATGCTTTTACTTCAGACTTCCAAAAACTCCCCACGAGAACCAAGGCAGTTTCTGCTTCACTATGGGTTCTGG
TATTCACCTTGAAATTCTTGGAGTCGTATTTTTTCTTGACATTATCTGTCAAGGATCCAGTCAAGGTTCTATCCCATTTGAGAATGACTAGGTGTCA
TGGTGATTCCATATTGGGTACACTTTCGTGCCGTTATCAGCCCACAATAACGCTTGCGTTTATATTCGTCACTGATTTGGTACTTTTCTTCCTTGAT
ACGTACTTGTGGTTCGTTCTCTGCACTTGCATGCTTTCCATAGTAATTGCCATAAAGAATGGCAATTCTATATTTACGCTGTGGAAGTCGGTGTTT
TCCCAGCTACCGGAAAGGTTGATATCCAAAACAGTCAACGTTGACAGCGTGGGAGACGCAATAGTGGCAGCCAGCCACATGTGGAATGCTATT
GTGTACTCCATGTATCGAGACCACTTGATATCAGTTGACCAAGCCTCAGCATTAATGTATCAACTCCCCGATACGGACTTTGTTCAAAGTTCAGCT
GATGTTAAGGCACCTATTAACTTCTTCAGTCACAATCATGCTATCCACTCGGATGAATACTTTCCTCAATTAGGTGAAGCAAAGAGACGAATCTC
ATATTTGCACAATCGTTAAGTTCGCCTCTCTGTAACGCAGATTTCACCACAGACGCTTGTCCAGCATTCACCGTATTGATCCCACACTATTCAGA

	AAGTATACTTCTTAGCATCGAAGAGGTGATCAGAAGATCTAAGCAAACCCAGATCACCTTGCTTGACTACCTCAAGAGTTTACTGAGCTCGGAC
	TGGACGAATTTCGTTCGAGACACTAGAGTCGCTGACGATGAAAAGTTCGGTTGTAGGTTTCCAATTCCTCTAACTTCGGAAGGTACAACAGACT
	ATGATAATTTGCCTTATGAGTATTACGGTTTCAAATTCGCAGACCCGGAAAGTACTTTAAGAACAAGGATATGGGCATCCTTACGCTCTCAAACTT
	TATACCGAACAGTTTCCGGCTTCATGAATTATAGACATGCGCTTGCCGAATTATACAAAGCGGAGCACGAAGATTGCATCAACCACATTCATCATT
	TGACTTTTGAAGACGAATTGAAAGCACTCATAGAATCTAAATTTACATTGCTAGTGTCGATACAACGACACAGCAAATTCTCAGAGTCAGAAAT
	GCAATCGTTCGAAATCATGGCTCAAAATTTCCCCACAATGAAAATATCAGTTCTCGAGGAGATCAAAGAGGGCGACAAACTGGTTCATTATTGC
	TCACTATTGGACTTGGCCAAAAAAGACGAAAGTAGCCAATATGGCAGAAAATTAAAAATCAGGCTTCCTGGGTATCCTATTTTGGGAGACGGCA
	AGTCTGACAATCAGAACACAAGTGCAGTGTTCTATCGAGGAGAGTACATTCAAGTCGTAGATTCCAACCAAGACAACTACTTGGAAGAATGTTT
	GAAAATCAAGTCAATGTTGTCTGAGTTTGAGGAGCTAAATTTGGCACCAGTAAGAGGAACATCCATGACCAGACCTCCAGTTGCAATTGTTGGC
	GCTCGAGAGTATATCTTTTCGGAGCAAGTTGGCGCCCTTGGAGACATCGCAGCAGGTAAAGAACAAACTTTCGGAACTATGTTTGGTCGAGCAC
	TAGCTTTCATGGAAGGCAAGTTACATTATGGCCATCCCGATTTCGTAAACGGGATCTTCATGTGCACTAGAGGCGGACTTTCCAAGGCGCAACGT
	TCACTTCATTTGAATGAGGACATTTATGCTGGCATGAATGCAATTGCTCGTGGAGGAAGAATAAAGCATGCCGACTATTTTCAGTGTGGAAAAG
	GCCGTGATTTGGGGTTCAATACAATCTTGAACTTTACTAGCAAAATCGGGGCAGGCATGGCTGAACAAACGCTATCTCGTGAGCAGTTTTATTTT
	GGAACTCGGTTACCTACCGATCGCTTGTTTTCCTTCTTTTATGCACATGTTGGGTTTCACATTAACAATGTGCTCATAATACTCTCGATTCATTTATT
	CCTGATATTCCTCTTTAACATAGGAAGTCTTCGAAATGAGAGCATTGTCTGTGATACCACAAGTGGACTCACTGAGCCTACTCCCATAGGCTGTT
	ACAATATCAAGCCAGCAATTGATTGGATCAGTCGCTACGTTCTTTCAGTCATAATATGTTTCTTTCTCTCGTTCACGCCATTGGTGATGCAAGAGT
	TTATTGAACGAGGAGTGTTGAAAACCGCTAAAAGAATCTTTTTCCACTTAATTTCACTTTCGCCGTTATTCGAAGTATTTGTTTGTCAAGTATACG
	CAAGTGCATTCGTGGATAATCGGTCGTATGGTGGTGCCAGATACATTAGCACTGGCAGAGGATATGCCATATCTCGAATTTCTTTCGCCACGCTTT
	ATTCGAGGTACGCATCGTTATCAATTTATTGGGGCTCACGCTTAAGTTTGATAATCATCTTTGCGTGCAGCACAGTATGGCAGATATCCTTGTTGT
	GGTTTTGGATCACTTGCTTATCCTTGTGCCTTTCACCGTTCATTTTTAATCCACATCAATTTGACCGAACGGAGTTCTTCCTTGATTATAGAGAGTA
	CCTTAGATGGCTTGGGAGAGGGAACTTTTCTCGATGCCGTAACTCTTGGGTCCGCCACGTTCGGTTTCAAATAATAAAGCTGACTGGGCTCAAA
	GGACCTGGAAGTAACGAGCTGGAAGACTTAGTTCACCCAATCTCCAAGTTCAGGTTCGCTATATGGAATTTGGCCAAGGCTATTCTCAACGTTT
	TGAGCTTCTTGGCTCCGTACATGTTCTTGAACTCACAAAATGGAGTAGCTGAGCCATCAAAAGTCAATCCTTTGATGCGGGTAGCTGTGATCGTT
	GCAATCCCACTAATCAGCAACATCATAATATTAGTTGTGCTCTTTGGTATCTCCATACTTCTAGGATCTATGATAAGAAACAAGCGTTTCGCGGATA
	CTATTGCAGCAGTTGCTCATATCTGGTCAATCTTGGCACATGTAATTGTCATCGAAATAACCTGGTACATCCATTCATGGAATCTTCCACGCAGTT
	TGGCGTGTATCTGTCTTGTGATCACCATTCAGCGGTACTTGATGAAGCTTGTGCTAAGTATGTGTATTTCCAAAGAGTCAGACAGGTTGGAAGCC
	AATTCTGCTTGGTGGTCAGGGCGATGGATTCAACACCATCTAGGATGGCACATCTTATCGCAGCCATTCCGAGAATTAGGATTGAAGTTAGTCGA
	GCTAAACTTCTTTGCATTTGACTTTCTTCTAGGGCATGTCTTGTTTTGCTGCTTGAGCACCATGCTCTTTATCCCGTATATTGGTCAATTACACTCG
	TTGATGATGTTTTGGTTTGTTCGTCACAACTCATTGAATGTGAGTGGAACACAGCACTTGCATAGTCGACAAAGGAAGCGCCAACGAAAACAG
	GCGTGGAGATATTTTTTCGTATTCATTGGAATCCTAGTGGGCATATCAGCATTACTTGTTGCTCCGTATCTTTTGAGAAAATACTGGGAATTCTTGG
	ATAAGTCGATTCCTACCTTTGCGCAGCCATTGTTCCAGCCTAGCAACCAGGATCAAAATGATACAGGACCCAGAGCACCTTCGAGCTTCTGGAG
	CAAAAAACCACCTCCAGCAACATGGTCTACCATATGGTGA
D_104046	ATGAAATCTGGTTTATTTGGATTAAGTTTGATTGCCGCAACATTTGCGAGAAATATTGTGTATCCAGAATGTTTTGAGGAACAAGTCAAGTTGGC
	ATTGAATGTTGATAAATTGGAACCAAGCGTGTCTCCTTCGGGGATCAAGTTGGTGTTTGTTTATAAGGCTCGTGACACCAGCGAAGAGAATTTG
	GATGGAGAAATCACCAAGGTGGTGGATGCTGAAACGCGGACCGCTGAGTCAACCGCTGGGTTGGCTACGTCGACTGCTGGGTTGACCGCTGCT
	CCAACTCCCGCCACTATTGTGACAAATTTCCAATACGGTACTACCACCTCCACCATCTATACCGCATCATCGACTCCAGCCTCCACCACATTTACG
	TCTGTCGAAAAATTGGAAACTACTCGAAGAAACCCTCTTGCAACGGGTGGAATCAAAATCTTTAAACCTCTTGCAGAGTCTATCGAGATTGCCA
	GCATAACCGATGGTGTGGTCTTTACAAACACCCTGTTTCCAACAAATGGACCAAAAATGCGGTATACACAGCCATCATTAACCACGAAATTTACC

	GTTGCATCTGCTGTTCAGCTGGCTGACAGTTTGGCCAACAAAGCGTTGACAGAAGTTGCCAACACTGCCTTAGAGACCAACGATACTTTACTGT
	CAAGGTCACTTGAACTCTCACTGGTTGAGAAGCATGTTATAGTGGTAGAGTCATCGTGTGAACTGTCCAGTTCTCTCGAGCCATCCTCGTTCAG
	TTCCGAGCCATCGAGTATCCCGTTCAGGTCCAAGCCATCGAGTATTCCGTTGAGTTCGGGCAGGTACTTGAATCTGACCTCTGCACTGGCCACTT
	CCGCTCCTTCATCTGTCTTGGTTGCACCCCACTATTCTGCCTCTTCAATTGCAAGCTCGCTGTCTTGCTACTCAAGCTCTGCATCGACTGGCTCGC
	TGTCTGGCTACTCAAGTTCTGCAACATCAGCTGGCTCGCTGTCTGGATATTCGAGTTCTGCATCATCAATTGCAAGCTCGCTGTCTGGATACTCA
	AGCTCAGCTCCTTCCTCATCGCCAGAAAGTGAGAGCTCAACCTCCGAGTCTTCCGTTGCTCTGTCGGCAAGTGTGACGGAATCCTCTAGCGAGT
	CAACAATTGCAACCTCGACCCAAACTTCGTCGGCTGCTTCGCCTCGATTATTTAGGGTCTTCTCTTCAACTTCGACTGCAAATGGTTTCACAGGC
	GATTTGTTTAAGGCAATTTCCACCAACGCCGTCTCAGGTAAATTTCCTAAACAAAGCTTACCATTGGCAATTCCATCTGGTGTGACAAACTCGGA
	TAAATACCAAACCAACAAGTTTTATGTCAATCTCTTTCTTGGTGATCAAACCGATATGATCTGGTCTTATCCCTATGGAATGCAGTACCTGAAGTC
	AACCTACTACGGATGGGCAGTACAACATACGATCCCCAGCGCTCGAGTATTTGGTAATGTCAATTCCAACAACAACAATCCCTCATACTTTTTCA
	ATCCAATCAACATCAAGGAATTGATTCTTTCAGCAACGTCTTTCACATCAAATCTCAAGATGTCAGTCTCCAACATGAAGGTAATGTCGGCTTTG
	GTCAAATTAGGACTGGCAACTAACTATATCGAGGTCCCTGTGGTGCAAGGTATGGGATTCGTTACTTCCATATACCATGGAAATCTCATACCTCAA
	ATAAACTCAGGAGTTGGAGTCAAAACTCTCGTGAAGGAAACTTCCTCCAACTTGTTGTCCAACATATTGAAATTTAGAGCAACTCTTTTCAGCG
	GCACTGAATATTTGATATATGTGACGTTTCCCAGTGGTACGTCCACCAGCGGATTCACATTTTCAGTTTCAAATTCGAATACAATCAAAGCATCTA
	AGAATATCAATGGTTTGATGATTCAAATTGCCGTGGCCCCATCATCGTCACAGGACAAATACTACGACCAGACGGCAGGAACGTATGTTACTGAA
	TCCAAAATCAAGGCGCATGGTTATGGTGGAACGACCGCTGAATATCGATTCTCATACATTAAAGCAGGTTCATCAAAATCCAACTTACCTATTGT
	GTTTTTATTGCCTCACCATGTTGACCTGATTGATGCAACCACCAAAAACGCTGCCACTGGTATCACCTTGTCATCCACAACTAAAGGAACAATGT
	CTGCTTATTTGGCTAGCGAAATCATCATGAATGAATCGCTAAACTACAACATTCAGTTTTTGCCTTGGGTTCAACAAATGGGTACAACTGCACCA
	TTTTACACGACGAATCAACTCAAGCTTCTTGCACTGGCGGCCAATACTGAGTTATCGGTAGATATAAAGACGATGGTCTTATCCATGAACTCAAA
	CTACTATTCAGGAAAGGTTCTTGATAAATATGCTTACATTCTCTTGGTTGTGAGTGATATCATTGGTGACGAAACATTAGCAAAGTCAACCTTGAA
	AATACTCAAGGACACTTTTGCAGTGTTCACCAACAATCAGCAATATTATCCCTTGATGTACGATACGAAGTTTGGAGGAATTACTTCGACTGCAT
	CACAAGGAGGTGATACTGGCGCTGAATTTGGAAGCGCATATTACAACGATCATCACTTCCATTATGGATACTTTGTACATGCTGCAGCCATCATTG
	GTTATGTCGACAAGAAATACGGAGGAACTTGGTATAAAGATCAACAGTTTTGGGTGAATGCGCTCATCAGAGATGTAGCGAATCCATCGCCAGA
	TGACAAGCAATTTCCCGTATTTAGAATGTTCGATTGGTTTGCTGGTCATTCATGGGCATCGGGGCTTTTCGCAGCCGGTGATGGTCGTAATGAGG
	AATCGAGTTCTGAAGATTACAACTTTGCGTACGGAATGAAGCTTTGGGGTAAAGTATCAGGCAATCAAAGAATGGAATCAACAGGAGATCTTAT
	GCTCGCGGTGATGAAGAGAAGTATGAACATGTACATGTACTATACTTCATCAAACTCGGTTGAACCATCACAGATACTTCCAAATAAAGTATCTG
	GTATCTTGTTCGATAACAAGATTGATTACACAACTTATTTGGAGCACCAAACGCACATCCCGAGTATGTCCATGGTATTCATATGCTTCCAATTAC
	ACCAGCATCTTCACTCATTCGAGGCAGTGCATACGTAAAGGAAGAATGGCAAGATCAAATCTCCACATTTATCTCCAACGTCAAAGACGGATGG
	GCAGGAATTTTGCGATTGAACCAAGCGCTCTTCGACGCATCTTCTTCGTACGCATTCTTCTCCTCGAGCTCATGGTCTTCTGCCTACCTTGACAA
	CGGTCAAAGTCGGACTTGGAGTTTAGCGTTTTCGGCCGGTGTCAGCAACGCATTGAGTTAA
D_104112	ATGAAGTGGGCCATTGGCGCTGCTGCCATTGCGGGTGTAGCTTTGGCTGATTCATCCAATAATTCAACCTTGACCACTGCCACTCCATCCGTTGG
	CAAGTCTTGTTCCTTCAAGGACTTTACTGCTACCAAGTCTGCCGACGTGCAGTCTGTCGCTGCATGTGCCACTGCCGTTGGTGACATCACCATC
	GAAGGTGACTCTTTCGGCACCATCGAATTGACCGGTCTCGAACAACTTTACGGTTCTCTTCAGGTGAACAATGCTACTCAAGCCACCAGCTTGA
	ACGCTCCTACCTTGCAATTGGTTTCTGGTCAATTGGCGTTGTCCGGTAACACCATCTTGTCTACCTTGAACTTGGCTCAATTGACCACTGTGGGA
	ACCTTGCACTTCAACGCTTTGCCAGCTTTGGAAAAAACCGGTTTGTCTGCTGGTATCACTTCTGCTGACGAGGTGATTATTTCCGACACTGGTTT
	GACTTCATTGGACGGAATCAATGTGTTCAAGTTGCAAACTTTCGACGTTAACAACAACAAGGATATCGAGACCATTGACTCTGGTTTGCAATCT
	GTCACTGAACTCTTGTCCATTGCTTACAACTCTGAAAAGGTGGACGTTGCTTTGGACGAATTGACTTCTGCCAACACCGTTTCGTTCCAGTCCA
	TCAACTCTTTGTCTGTTGCCAACTTGACCAAGATCGGCGACTCTTTGTCGTTCGACTCCAACTCGTTGGACAAGATTGAGTTCAAGCAATTGAG

	CTCGATCGGCAAGTCTTTGACCATTCAAAAGAACGACAACTTGGAGGAAATTGACTTCCCCAAGTTGAAGTCGATTGGTGGTGCTTTGGTGATC<br>CAACTGAACGACGAATTGAAATCTTTTGATGGCTTGCCAAAGTTGGAAACCATCGGCGGTTCCGTCAACCTCAAGGGTAAGTTTGACAATGGTA<br>CTTTCGAGTCTTTGCAAAGAGTTGCTGGTGGTTTCAACTTGGACTCCACTGGAGACTTGACTTGTCTGGAATTCAACAAGTTGAACAAGGACG<br>GAGACATCAAGGGTGACAAGTTTGTGTGTAAGGCTGCTGACGAGTCGTCATCTTCTTCGTCTTCAAAGAAGGGCAACTCCAATGGTACTGCCA<br>CCAGCGATGACTCTTCTTCTGAGACCAGCAGCTCCGGTGGTTCTGGTTCTTCTTCTTCGAGCTCCAAGAAGAGTGATGCCAACTCTGCTGGTCT<br>TAACTTGGCTTCCATTCTTGCCGGCTTCGTTGCCCTTGGTGCCACCTTATTTTAA
D_101126	ATGCTTTACACTTCGATTTTGGCAGCGTTGACCGCAGCCACCGCCGTTGGGGCCGCTAGTGACAAATGTTCATTCTCTACCACCGTCAAGGCAG<br>CCACTGCTATTTCTGACTTAAATTCTTGTGAAACCTTGGATGGTACTATCAAGATTACTGGTGACGATTTGGGTGATATCGACCTCAGTGGGGTCC<br>AAGAAATCAAAGGAGATATCAACTTTTTCAACTCATCTTCTGTCACCTCCATTAATCTCAATCAGTTGAAAAAAATCTCGGGTTCGTTGGCAGTT<br>AATGCTTATACCCAATTGCACAGCATTGACTTCACCAGTTTGAGTGAAGTCGAGAAATTGTCTTTGATTTCATTGCCATCATTTGCCATTCTCAAC<br>TTGAACACTGGTGTTTCGAAAGCAGGTTCCATCGAGATTTCAGACACTGCTCTTTCGTCGTTACAAGGTCTTACCAACTACGACACCGTCAAGA<br>GCTTGAACGTAAACAACAACAAGAACATCACCTCCATCGACTTGGCATTGCAAACTGTCGACGAGGACCTCACTTTGAGTTTCAACAGTGACG<br>ACTGTGAGGTCAAATTGAACGAATTGATCTGGTCTTCCAATTTGACCATCCAAGATGTCAGTGACTTTTCTGCCTCCAACTTGACCGCCGTCAAT<br>GGTACTTTGAACATTGCTTACAACAAGTTTGACCAATTTGACCTCAAGGAATTGACCAATGTAGGCGGATCTGTTCTCGTTTTCGCTAATGACGA<br>GATGACTTCGTTCGACTTGAGCTCCCTCAAAAACATTGGAGGAGAATTGAGAATCTTCAACAACACCGAGTTGGAAGACATGAACGATACTTTT<br>AAGAAGTTGGCCAAGGTTAAAGGTGCCGTCAACATTAACGGTGCTTTCCACAACTTGACCATGCCTGGGTTGAAGGAGGTGGATGGAGACTTT<br>ACCGTTGTTTCCACTTCTGACGAGTTCAGCTGCCAAGATTTCAACAAGTTGAAAAAGAATGGTGACATTGAGGGACACAACTACAAGTGCTCT<br>GCCCCCAAGAAGGAACAATCTTCCAAGTCCAACTCTTCCAAGTCGAGCAGCGGATCTGGATCTTCTTCCTCTGACAGCTCCGATTCCTCGTCCT<br>CGTCGTCATCTGATTCTGGAAAGAAGAAGAGTGGATCCACCAAGACCATGGCTGGAATGACATTTGTGTTTGCCGTTGTGGGAGCAGTGATTGC<br>CATGGCATAA
D_102974	ATGAGTAAAACAGGCCCGGAAATCGACCAAAGATTTAGCATGAGAAACGGTGTCGAGTCCTCGGATGTCAGCATGAGAGTAGGGAACGTTAGT<br>GACATCGATGAGGAGTCTACGGGACCCATGGAGGTGCCAGAGCCCAAGAAAAACAAAAGTTTTTGGAGGTACACTTTTCCCGCTGCATTTTCA<br>TCTCTCGGTGCCATTTATGGAGACCTTGGAACATCTCCATTATATGTGCTAAACTCGGTCAAATACCCTCATAAAGAACCCACTGAACGAGATATC<br>ATCTGTGCGGTTTCGGTGATCTTTTGGGTATTCACGCTCATTGTTATCGTCAAATATGTGGCGATTGTGCTTTTCTTTGGTCCCAATAATGGTGAAG<br>GTGGTCAGGTAGCCATCTACGCCAAAATCGCCCGACACCTTAAAATCGGTCCTAAAGGTGTAACCATTCCTGGAGCACCGGAAAAAACCGATCT<br>CGAGCTTCTTTCTCGACAAGAAACCGTGCTGAGCTTTGTTTCAAGTACCAATAAAGCGTGGAAACAAAATCCGACAGTGGTGAAAGTGGTCTC<br>GTTTGTGGTTCTAACAGCATGCTTTCTTGGGTGCTCGTTGATCATTTCCGATGGTCTTTTGACTCCCACAACTTCGGTGTTGAGCGCTATAGCTGG<br>TATCCAAATTGCAAAGCCAGACTTTGACAATGTCCTTGCGGTTTCTGAGGTGGTTCTTTTGGTGCTTTTTTGTATCCAGCAGTTTGGATCCCACA<br>AGATATCGTTCACTTTTGCACCCATAATCACCCTTTGGCTTTTCGGCTTGATTATCTGTGGATTGTACAATATCATCAAATATTATCCTGCCATCTTC<br>AAGGCTATTTCTCCGCATTATGCCATTGAAATTTTAAAGGCGGGTGGAATCGACGTCTTTAGTGGTTGCATGTTGGCTATAACTGGAACAGAAGC<br>TATGTTTGCCGACGTGGGACATTTTGGTCGAGCACCGGTTCAGCTCGCTTTGACTTGTTTCGTGTATCCAGCATTGATGTTGTGCTATTTTGGACA<br>GGCTGCGTACATCATTCATCATCCAAAAGCACTTTCAAATCCATTTTTCTACTCGATTCCTGGAGGCACCAATAGTGCGCCATACTGGATTATGTT<br>CGTGTTGGCTACACTCAGTACCATCATTGCCAGTCAGGCACTCATCTTGGGAGTTTTCAGTATTCTTTCGCAATTGATCAATTTGGACTGTTTCCC<br>AAACTTTACTATAATTCATGTGTCCAAATCGCATGCCGGTAAGGTGTATCTTCCAATGGTTAACTGGATGCTCATGGTGGGTGTGCTCTGTACCAC<br>TGCTGGGTTCAAAAATAGTAACAATGTCACCGCTGCTTATGGATTGGGTATCACGCTCGATCTTTGTTTGACGACAATTTTGTTGACTTTATGCTT<br>CATTTTCGTATATCAAGTCAACATTTTTGTGCTGGCTTTCTTCCTTTTGGTGTTTCTTCCGTTGGAGATAGTTATGGTGATTTCCAACTTGAAGAAA<br>ATCGAACATGGAGCATGGTTCCCCATCATGATGGCTGGAATCTGTTTCTCTTTTCTTTGCTTTTGGCGGTGGGCAAGAGCCAGAAAAGTCGACC<br>ACGAATTCAGTTCGAGGGCTCGTATAGATAATGTTTTTCCATCTTTGAGACGGACTGCTCAAACCGTCGATTTGGGCCGCGGAAGAAGCCCTAC

	AAGAAAAGACGAAGACGAAAGCCGTGAAGAATCTGTGGCAGAATGGAACGAGAACTTGATTGTGAATTCAAAGTTTGGTGAATTGGCATTGA<br>AGACATACGACGGAGTGGCTATAATCCATTGTGAATCATCGTACCAGAATCTCATGTCTCCAAATACCGTTCCTGAATTGTACCAAAGGGTGGTT<br>TCGTCGTTTGCTTCGTTGCCGAGAATTGTGATTTTTTGCTCCAAAAGAGCGTTATCTGTTCCTGTGGTTCCACAAGACGAACGAGTGCTTCTTGG<br>ACCTACCAAAATCCAGGGCCATTTTCGGTGTGTCTTGCGATATGGGTTCACCGAAGAGATGGTTATCGATAAAGATTTGATGCAACATATTCTCA<br>AGTCTGTGCCTGGATATGTTGAGTTGAACGATAGTCCTCATCGAGACCAGATTCCGGTTCCAGTACTTCATGTTTTCGACAAGAGTGTTGTCAAG<br>TCTCACACCTACTCGAGCAATCCCACTCGGAATATCCTTAGAAAAGCCGGAAGACGGGTGCGTATTTTTGCCATAGAGCATATCTTCAGTCCTAT<br>CACTTCGATCTTCAACTTTCACGGGCAGTACTTGAAAATAGAGGATGAAGCCGAAGAAACACAGCGCAAGTTGTTCGTTGGAGGAGTAGTTAG<br>AATCTGA
D_104268	ATGGAGCACTTTGATCCTCGTCGTGAGATAGACAAATACTCGCTTGATCTGTCTACTTTTGACCTTATAGATGTGGGAAAATTTCACAATTTGAAA<br>TGCTCGACTGTGTTTGATTATATGTTCACATGGCTGCTTCTAATCCTTGGTGTGGTACTTCTAGGCGTGGATATTTACACTTGTCTCAACATTCTTG<br>TTTTTCACCGCTGGAGTTCCGACGACTATAAACCCTATGCATACTCAATTGCCAAATGGATATTTACCGGGTGTATTATTTTCCAGTTTGTGCTTCT<br>CTTGTACCACTGGATATGGGCCATCCATACTTATAGGACCCGAAACATAGCATTGGCATACGTGAACAATATTGCTCGTCACTTGTACACGATTAA<br>ATCTTACGACTATCATTGTCTTTTCAACCTGGTGGAACATGACAATTTCTTTGATTGGGCATGTTTTCTTGCATACTTCGAGATGGACTCAGCTTT<br>GCAAATTCTCGTCGCCGATACTCCTCGTCAAGTAATCAATATCTTGACCCTTCGTTACTACGCTACCAACGAAAATTCTTCAAATGATATCATTCA<br>AAATATCAAGCAGATCGCTACATCTAACATTCGGCTCTCGGTGATCTTGTCGTTTATGCTTCTTTCCGTGGCCATTTGGTCCATTTTCTTCTTTCGT<br>TTCGTGTTTGGGATGCTCTGCTACATTCCATGTTTGATGAAAATTCGCAAAAAGGGCCACACTAGACTCAAAAAATACTGCTGTTCCGTTGTTAA<br>TCTGCACGTTCGCCGGTTCGTCTACAAGCATCACAAACCACGCAGCAAATTACTTGAAGAAGGTATTCTCGATTTAAAGGAAATCAATGAAAAT<br>CCTCTTCTCAACTCAGCGTCCACCACTGCCACATTTGACTCGGCTTTCCAGTACAAACCCGAACCAGCCAAAACTTTCCAGCCAATGGACCGTT<br>CTTACGACACACTTCCACGCTACGGCTCAAGACAAAACACTTATGAATCGTTACCACTCCAGAACATGCCAAATATGCAACGCCGTAGACCACC<br>ATATGATCCATTTGGCGACGAGAACAAGATTTCGAGCAAAGCACATTTGATGCACCAGGATCCTTTCGGCGATCCTAAACTTGAAGATAATGATA<br>TGAGCGACACAGAAAACGTATACGAGCTCTATAGAGGTGTGGAAGTCGAGCCACCTATGCATAAACGGTATAATGGATTTGATCCACCACCCAG<br>AACTGGTTCAGCTTCTTCGCTCACATTTTCTACAACAGGACCTTTTGAGCCAGTATCACAAACCCATACTGACCCGGTTGCACCGCCAGCTTCC<br>ACTCCGTCTATTTCTGGGCCTATAACTCGAACGGGGACAGCACCATATCCACCAGAAGAAACAAGCTCTTTGTTGGGAGAGACATCGGAAAGT<br>GACTTGCATTCTAGGGAACTGGAACCAATGGAACTGGAACCGGAATCGAAACCAAGGGAAGCCCCATACCCCATACGCGAGTCGTACGCTCTC<br>CCAAGAACCTCTGACGAATACTACAACAGGTAG
D_101005	ATGACAGAGAACCCGTTCGATGTGGAACAGGTTCTAAAACCACCCCAACGTGTCAACTCGGCTGCAAACCGCTATTGCACCCAGTCGGTGGAG<br>GACACGGTGCTGGAGTTCAAAACCGATCCTCACAAGGGGCTTTCAGACAATCAGGATATTCTCAACCGTAGATCCATACACGGTGTCAACGAGT<br>TTGCCGAAGATGAAGAGGAGAGTCTTGTGAAAAAATTCATCGCAAGCTTCTATTCTGACCCATTGATCTTACTTTTGATCGGGTCTGCTGTCATT<br>TCATTTTGGATGGGAAACGTCGACGACTCCATATCTATCACCTTGGCAATCACTATCGTCGTGACTGTAGGTTTTGTCCAGGAGTACCGTTCGGA<br>AAAATCGCTAGCTGCATTGAACAAATTGGTTCCAGCAGAAGCCAACTTGACACGCAATGGTAACACCTCTCATGTTCTAGCATCGACCTTGGTT<br>CCTGGAGACGTGGTCCACTTTTCCCAAGGTGACAGAATCCCCGCCGATATCAGAGTAACGGACGCAGTCCACCTCTCCATCGATGAGAGTAATC<br>TCACCGGCGAGAACAGACCGGTAATAAAATCACCAGAAGCCATCAAATCAGAAGCAAACGGTCTTATTCCAATCACTAACCGTCCGTCTGTGGT<br>CTACATGGGAACCTTGGTTCGCGATGGCCATGGTTCTGGAATTGTCATAGCAACCGGTTCCAAAACGGAATTCGGTGCCGTTTTCGAAATGATGT<br>CAGAAATAGAAAAACCCAAGACTCCGTTACAACAAGCCATGGATAAGCTCGGAAAGGATCTTTCCATCTTCAGCTTTTGTGTCATTGGAATCAT<br>CTGCTTAATTGGTATTACCCAGGGTCGCTCGTGGTTAGATATGTTCCAGATTTCCGTTTCTTTGGCCGTGGCAGCCATTCCCGAAGGTCTTCCCAT<br>CATTGTCACAGTCACTTTAGCCCTTGGTGTGCTTAGAATGGCTCGTCATAGAGCTATCGTCAAGAGATTACCCAGTGTCGAAACATTAGGAAGTG<br>TCAATGTAATTTGCTCTGACAAAACTGGAACTTTGACTGAGAACAGGATGACTGTGACGAAAATATGGTCGACTGATTTTGAAGGTACGTTCAA<br>TTCCCCATTCTTGGTGGTTGAAAGGCTCGATGATAATACTTTACACCACAAGCTCACCAGCAACATTCGCAGAATATTAGAGGCTGGCAATATCT

GTAACAACGCCAGGTATTCATCTGAAAGCGAGAAGTTTGTTGGAAACCCTTCCGATATTGCCTTTGTTGAATGCTTACCACACTTTGGTCTTGAC
GATACTCGTGGTCAAAAAGTACGTACTTACGAATTGCCCTTTTCCTCAAGAAGAAAGTATATGGCAGTATGTGCTCATTCTGGCGACACTTCGAG
ATCTGAGACTTTTGCTAAAGGTGCAACTGAGCAGATTCTTGCACGTTCCACGAAATACTACGACTCTAAAGGTGAGGTAAAGCAACTTAGTGAC
GAAGTAAGAGACGAAATCAACCAGAGATCCAACACTTTGGCGGGAGAAGGGTTGAGAGTTTTGGCCTTGGCCAACAATACGCAAAAGTTCGG
TGACGAGAAACATATCGAGAGTGAACCTACCGATTTAATTTTTTGCGGGTTGATAGGAATGAAAGATCCTCCCCGTCCTAATGTGAGCAAATCGG
TTGCACGTCTCATGAAGGGTGGAGTACATGTGATTATGATTACTGGTGATTCTCCAAGCACAGCTAAGAACATTGCCAAGCAGATTGGAATGCCA
TTACATACCAACGATTCTGTCATGACCGGCGACCAACTCGACAAGTTATCCCCCGAAGCACTTTCGAATGCAATCCACAACGTTTCGGTGTTTGC
TAGAACCACTCCAGAGCACAAAGTCTTGATTGTCAAGGCGCTTCAGGCTAGAGGTGATATCGTCGCCATGACTGGAGACGGAGTCAATGATGC
CCCAGCATTAAAGCTCGCCGATATTGGAATTGCCATGGGAAAAAATGGAACCGATGTTGCCAAAGAAGCAGCCGATATGGTGTTGACGGATGAC
GATTTCTCTACGATTTTGAATGCGATCGAAGAGGGTAAAGGTATTTTCTTCAACATTCAAAACTTTATCACTTTCCAGTTATCCACCTCGATTGCC
GCGTTGACGTTGATTGCTCTCGCTACGTTCTTTGGACTTCCAAACCCTTTGAATGCTATGCAAATTTTGTGGATCAATATCTTGATGGATGGCCCT
CCCGCCCAGTCGCTCGGAGTTGAACCCGTTGATCATGAGGTGATGAACAAGCCTCCCAGAAAGAGAAACGACAAAATCTTGACCAAAGCTGT
GATCAAGCGTGTTTTACAATCTGCCACTATGATTATTCTTGGTACGCTTCACATCTTTGTCAAAGAAAGAATTGATAATGAGGTGACTGCCAGAG
ATACCACCATGACTTTCACCTGTTTTGTAATGTACGACATGTTCAATGCTTTGGCATGCCGTCACCAAACCAAGTCTATCTTTGAACTCGGACTTA
AAAACCAAATGTTCAATTTTGCCGTGGCCGGATCACTTCTTGGTCAATTGTGTGCCATTTATGTGCCATTTTTCCAGTCTGTCTTCCAAACCGAG
GCATTGTACTTATCAGATCTTGGAAGCCTTTTCTTGTTGACCAGCAGTGTATTTTTAGTTGACGAGGCAAGAAAGTGGTATAAGAGAAGGCAGTT
TACGATGAATGGAGTCAGCTACAGCGTATAG

D_103758 ATGGCTCCACAGAATCCAATAGAAAGGGCGCAAACAGCGTCCAAACATTTACAACTACCCGGATCTACCTCGGATAATGAAGACAATATCAGTG
TTTCATCAGCCACACCTCTCACAGATAATTCAGGATTAACTCCAGAAGTGTTAACGGAACTTCACGATCCCAAATCGCTTCGCAAATTGCATGAA
CTTGGTGGGATCAAACTGTTACTGTATGGTCTTGAGACTAATTTGCTGAGTGGAATCGATACCCATAGAGATCTTAAACATAGAGAAGAATTGTT
CGGAGAAAATAGAATACCGGTGAAAGCCCAGAAAAACTTCTTTCGTCTATGTTACGATGCCATGAAAGACAAGGTGTTGATCATGTTGACTGTG
GCTGCGGTTATATCCTTAGCATTGGGCTTATATGAGACCTTTGGTGAAGGTCCTTTGAGGGACGATGAGGGCAAAGTTTTGCCCAAGGTGGACT
GGGTGGAAGGCGTGGCCATTATTACTGCCGTTGTAATTGTGGTGGTGGTTGGTGCAGCCAATGATTATCAAAAAGAACGACAGTTTGCTCGTTT
GAATGCCAAGAAAGAAGACCGCGAGTTGATCGTGGTAAGAGACGGAGCCCAGAAAATGATCTCCATTTACGACCTTTTGGTCGGTGATATCATC
AACTTGCAAACAGGTGATGTTGTTCCTGCTGATGCCATCTTGATACTGGGAGACGTTGAATGTGACGAATCTGCTCTCACTGGTGAATCTCATAC
CATTAAGAAGAAACCCGCTGGCGAGGCTATGGATTTCTATGAGGCACAGCTTCCAACAGATGAAGATTTGGGCTCGTCTACCATCAAGTTCAAG
GACCCTTATCTTATTTCTGGTGCAAAGGTGCTCGAAGGTTTGGGATATGGAATGGTCACTGCAGTTGGCCCCAATTCCATTCATGGAAGAACAAT
GATGAGTCTTCACACCGACGCAGAAACAACCCCCATGCAAGTTAGACTCGACAACCTTGCCGAGGGAATCTCCAAGTACGGTTTTTTGGCCGC
ATTGGTCCTTTTCATCGTTTTGTTCATCCGCTATTGTGTCAATATTGCTCCAGGAGGAAAATTCAACGATATTCCTGGTCCGCAAAAGGGAAAGA
AGTTCTTGGACATTTTGATTACTGCTATAACAATTATTGTCGTTGCGGTTCCCGAAGGTTTGCCATTGGCAGTGACTTTAGCGTTGGCTTTCGCCA
CTACTAGAATGGCTCAGAACGGTAATTTGGTCCGTGTGTTGAAGTCATGTGAGACCATGGGTGGTGCTACCGCTGTGTGCTCTGACAAAACCGG
AACTTTGACTGAAAATCGTATGAGAGTCGTGAGAGCATATTTTGGACTGTCCGAGTTTGACGATACTTCTGGTGGCCATGGTCCATTGTCTTCAG
AAGTGGTGAATGAACTTTCAGAAGAACTTAAGGTCTTTCTTTGTACCAACATCACTTTAAATTCCACTGCATTTGAGAACACCGACTACGACGA
GAAGAAAGCTCTTATGGCAAGACAAAAACCACAGAGAAAGTCATTTATCCGCCAATTGATGCAAAACCCCGGAAAGAAACAACAAGAACGAC
AGGTTGAGCTTGGAGTGGTAACCGAACCTTATTTGGGAAACAAGACGGAATCTGCCTTGTTGATCCTTGCTAACAAAGTTTTCAATCAGTTTGC
AACAGACAACTTGGAGACCCAGCGGAGCGCCAACCACGACAAGATAGTACAGATCATCCAGTTTGAAAGTTCGAGAAAATGGGCTGGAATAG
TAATGAAGATAGACAATGGGTTCCGTCTTTACGCTAAGGGAGCGGCTGAAATTGTTTTCAAGAATTGTGGGTACCTAACCAATGTCGATGGTACT
ACGGTGTCCATGGATCGGTCACAGAGAGACGACGCTTTCAGCAAGATTGATGAGTATGCCAATGATGCTTTGAGAGCCATTGCACTTGCTCACC

GCGACTTCATTGGAATTTCCAACTGGCCTCCACCAGAACTTTTAGAGGAAAACTCCAAACAAGCCGACCCTAAGAAACTTCTTGCTGTAGGATC
CACTATTCCAGAAGACCAAAGACACTTGGTTTTGGATGGAATTGCAGGTATTCAAGATCCGTTGAAAGACGGTGTGGCACAAGCAGTTCTTCA
GTGTAAGGAAGCTGGTGTCACTGTTAGAATGGTTACCGGAGACAACTTGAATACTGCAAAATCCATCTCCAGAGCATGCCATATTCTTACTCCTG
ATGATCTTTCGAATGACTACGCATACATGGAAGGACCAACCTTCCGCAAGTTGACGGATGCGGAAAGAACGCGGATTGCGCCTCGTTTGAAAGT
ATTGGCGAGATCTTCTCCAGAAGATAAACGGGTTCTTGTGGAAACATTGAAGAAAGCAGGAGAGGTTGTTGCTGTCACGGGTGACGGTACAAA
CGATGCTCCTGCCTTAAAGTTGGCAGACGTGGGATTTTCTATGGGAATTGCTGGTACTGAAGTGGCCAGAGAAGCCTCTGATATTATCTTGATGA
CAGACGATTTCACCGATATTGTCCAGGCCATCAAATGGGGAAGAACTGTTTCGACTTCGATCAAGAAATTCATCCAGTTTCAGTTGACCGTCAAT
ATTACTGCCTGTATCTTGACGTTTGTGTCCGCTGTGGCATCTTCCAATGGCCAGTCGGTGTTGACTGCTGTGCAGTTGTTGTGGGTGAATCTTATC
ATGGACACTTTAGCGGCGTTGGCGTTGGCGACCGATAAGCCAGACGACTCGTTTTTGAAGAAAAAGCCTGCTGGTCGTACTGCTCCTTTGATTT
CGGTTTCGATGTGGAAGATGATACTTGGACAGTCAGTCACTCAGTTGGTTATCACGTTCATTCTTCATTTCTGTGGCAGAAGATTTTTCCATGGC
AATAATCACATCGACAACCATCAGAACAAGCAATTGGACGCCATGACTTTTAACACATTTGTGTGGTTGCAGTTCTGGAAATTGGTCGTCACGA
GAAAGTTGGACGAAGCAGATGGCATTCGAAAGGTTCGGGACAGACTCACGGCCAACAACTTGAACTTTTTTCAACACTTGTTCCGCAATTGGT
ACTTTTTGGGAATTGCTCTTCTCATCGGCGCAATGCAAGTATTGATTATGTTCGTCGGTGGTGCTGCATTCAGCGTTGTTAGACAGACCCCTGGT
CAATGGGCTACTGCAATCATCTGTGGATTTATATCGATTCCCGTCGGATTGGTCATAAGAATAATCCCTGATCACTGGGTGGTGGCCATTTTCCCA
ACCAGAGCCTTCAAGATCTTTATTTATTACGCTGGGTTTCTGTTTTTGAAACGTAAGAAGAAGGAGGATCTTGAGAAAGCTGAACACGATGACC
TTAATGACACTAAGATGTCGGACTTGTCCGCACAATCATGA

D_101154 ATGTCGATCCCCAACCCCGTCAGCTCGGTGTCCAAGGTTTACACGGATGTTTTAGCTTCGAAACCACAGTCGTACTGGGATTATGAAAACATAAA
TATCAAATGGAACTCGCAAGACAACTACGAAATCATCAAAAAGTTGGGACGTGGAAAGTATTCGGAGGTTTTTTTAGGAGTGGATCTTAAAAA
AGGTGAAAAATGCGTTATAAAAGTGTTGAAACCGGTCAAAAGAAAGAAGATCAAGAGAGAAATTTCCATTTTGAAGAACTTGGACGGCCCCA
ATATCATTGGGCTTTTTGATATTGTAAGAGAACCCCAGCTGAAAACACCCGGACTCATCTTTGAGCACGTCAACAACATCGACTTTCGCACCCTC
TACCAGTCTTTCACCGACTATGACATTCGGTTCTACATGTACGAATTGTTGCGAGCGTTGGACTACTCGCACTCGATGGGGATCATGCACCGTGA
CGTCAAGCCCCACAATGTTATGATAGACCACGAGAAGAAACTTTTGCGGTTAATTGACTGGGGTTTAGCCGAATATTACCATCCAGGAACCGAAT
ACAATGTGCGAGTTGCATCGCGGTATTTCAAGGGACCGGAATTGCTCGTGGATTTCCGTCTCTACGATTACTCTCTTGACTTGTGGTCGTTCGGG
TGCATGTTGGCGTCAATGGTTTTCAAGAAAGAACCGTTTTTCCACGGAAAATCGAACACCGACCAATTGGTGCAAATTGTGCGTGTGTTGGGAT
CGGACGACCTACACAAATATTTGCAGAAATACGGGCTTGTTTTGAGCGAGGAGTACGAAGATTTGGGATACTACAATAGAAGACCTTGGAAGCG
ATTTGTCAACGAGAACAACCAGCATCTTGTCAGTGACGAGTTTCTTGATTTCATCGACAAGTTGTTACGCTACGACCACCAGGAGAGATTGACG
GCCAAAGAGGCTATGGCACATCCGTACTTTGACCCAGTACGGCCCAAGTAA

D_103937 ATGGTTTTGCCTCACGAGCCCGAATTTCAACAAGCTTACAACGAATTGGTTTCCGCTTTGGAGGAATCCACCTTGTTCCAAGAACACCCTCACT
ACAAGAAGGTGATTCCTGTTGTTTCAGTTCCCGAAAGAATTATTCAATTCAGAGTTTCATGGGAAAATGACAAGGGAGAAATCGAGGTGAACA
ATGGTTTCAGAGTCCAATTCAACTCGGCTCTTGGTCCTTACAAGGGAGGATTGAGATTCCACCCAACCGTCAACTTGTCGGTGTTGAAATTTTT
GGGATTTGAACAAATTTTCAAAAATGCCTTGACCGGTCTCTCCATGGGTGGAGGTAAAGGTGGATGTGACTTCAACCCCAAGGGAAGAAGTGA
CGCTGAAATTAGAAGATTCTGTGTTGCTTTCATGAGACAATTGGCCAGATACATTGGTGCCGACAGAGATGTTCCTGCTGGAGACATTGGTGTTG
GTGGTCGTGAAGTTGGTTACTTGTTCGGTGCCTACAAGCAAATGCAAAACAACTGGTCCGGTGTTTTGACCGGTAAGGGTTTGAGCTGGGGTG
GTTCTTTGATCCGTCCTGAAGCCACCGGTTACGGTACTGTCTACTATGTGGAGAAGATGATTGAAAAGGCCACTGGTGGAAAGGAGACTTTCAA
GGGTAAGCGTGTTGCCATTTCTGGTTCTGGTAACGTTGCCCAATACGCTGCTTTGAAGGTTATCGAATTGGGAGGAACTGTGGTTTCTTTGTCTG
ATTCTAAGGGAGCTCTCATTTCACAATCTGGTATTGTGCCTGAGCAGGTTGAGGCCATTGCTGCCGCTAAGCTCAAGTTCAAGTCATTGGAAGA
AATCTGCAAAGAATCTGCTTCCATTTTCTCGGGCAAAACCGAGTACATTGCCGGTGTCCGTCCATGGACCAAGGTTGGTCAAGTTGACGTAGCA
TTGCCATGTGCCACCCAAAACGAAGTCAGCGGTGACGAAGCTAAGGCTTTGGTTGACGCTGGATGCAAGTACATTGCTGAAGGTTCCAATATG

GGATCCACCGTCGAGGCTATCGACGTCTTCGAGGCTAACAGATCCAAGAATGTTTGGTATGCTCCAGGTAAGGCCGCCAACTGTGGTGGTGTTG
CCGTTTCTGGTTTGGAAATGGCCCAAAACTCTCAAAGAGTTTCCTGGACTTCTGAACAAGTTGACGAAAAGTTAAAGAACATCATGTACACCT
GTTTCGAGAACTGCTACAACACTGCTATCAAGTACTCTACCGAGAAGAGTGCTTCTGGCTTGCCATCTTTGTTGCAGGGTGCTAACATTGCTGGT
TTCATCAAGGTTGCCGATGCCATGTTTGACCAGGGTGAGGTGTTTTAG

D_102983 ATGTCCCGTTTTCTAGCGTCGAAATTCGCCAAGACGGCCCTTGCAACGACGGCCGTTATTGGTGGTTCTGTTGTGTACGTGGACTACATCAAACC
ACCCGCAGTGCCTGAGTTGGTCACTTCGTACAAACCTTTAAGAAAAGATTTGCCTGCTCCTCCAAAGAGAGAAGAGCTCGTTTCTCGGTTGGA
AACCACCCCCAAATTCGATGTTCTTGTGATTGGAGGTGGTGCCGTGGGAACTGGTACCGCGTTGGATGCTGCTACAAGAGGCCTCAATGTTTGT
TTGTTGGAAAAAACCGATTTTTCTTCGGGTACTTCTTCAAAATCTACAAAAATGGCCCATGGTGGTGTCAGATATTTGGAAAAAGCCATTTTCCA
GCTCTCTAAAGCCCAGTTGGACTTGGTTATCGAGGCGCTTAACGAAAGAGCAAACATGTTGCGCACGGCTCCTCACTTGTGTTCCGTTTTGCCC
ATCATGATCCCCGTCTACAAGTGGTGGAAAGTGCCTTATTTCTTCATGGGTTGCAAAATGTACGATTGGTTTGCCGGTCACCAGAACTTGCGGTC
GTCCACTGTTTTCTCCAGAGAAATGACCGCTGCCATTGCTCCTATGATCGACGATTCCAACTTGAAAGCTGCATGTGTGTACCACGACGGAACTT
TCAACGATGCCCGTATGAATGCTACTTTGGCCATCACTGCCGTCGAGCATGGTGCCACCGTTCTCAACTACTTTGACGTGGTCCAGCTCGTGAA
AAATGAAGGCAAAATCGAAGGTGTGCGTGCTGTCGACAGAGAAACCGGTAAAGAGCATCTCATCAAGGCTACTGCTGTGGTGAATGCCACCG
GTCCTATGGCTGACAAGATTTTGGAAATGGACGAAGATCCTCAGGGCTTGCCTCCAAAGATCCCACAACCACCAAGAATGGTTGTTCCTTCGTC
TGGTGTCCATGTCGTTCTTCCTGAATACTACTGTCCAAGAGATATGGGAATGTTAGACCCATCCACCTCCGATGGTAGAGTCATGTTTTTTTGCC
ATGGCAAGGAAAGGTCTTGGCTGGTACTACCGACACCCCATTGAAGTCTGTCTCCGAAAACCCAGTCCCATCCGAAGAAGAAATTCAAGATAT
CTTGAACGAATTGTCCAAGTACATTGTTTTCCCTGTGCATAGAGAAGATGTGTTGTCTGCCTGGTCTGGTATCAGACCATTGGTTCGTGACCCTT
CCACTGTCCCCAAGGGTCAAGACCCCACCACCGCTTCTACCCAAGGCCTTGTTCGTTCCCATTTAATCACCCAGTCTGAAACAGGATTGGTGAC
GATTTCTGGAGGAAAATGGACCACTTACCGTGAAATGGCTGAAGAAACCGTTACT

D_102964 ATGACTACCACTCCTTACTCTATTCCAAAGCCATTTAAAGTTTGCATTATCGGTTCCGGTAATTGGGGAACTGCTGTGGCCAAGTTGGTGGCAGA
GAACACTGCAGAAAAACCACAAATCTTTGAAAAAAATGTTCAAATGTGGGTTTTTGAAGAACAAATCGAGGGTAAAAATCTTACTTCCATTATC
AATGAACAACATGAAAATGTCAAATACTTGCCCGGAATCAAGCTTCCTGAAAACTTGATTGCTAATCCTGATGTGGTATCCACTGTGGAAGGCG
CTGACCTTTTGGTGTTCAACATTCCTCATCAATTTCTTCCTCGTGTTTGCAAGCAATTGGTGGGTAAGGTTTCTCCAAATGCCCGTGCCATCAGTT
GTCTTAAGGGTCTCGAGGTTAACTCTGAGGGATGCAAATTGTTATCCCAATCCATCACTGACACTTTGGGAATATACTGTGGTGTATTGAGTGGA
GCCAACATTGCTACTGAAGTTGCCAAGGAACGTTGGTCGGAAACCACCATTGCATACTGTGTTCCTCCTGACTTCCGTGGAGCCGGTCACGACG
TAGACGAATACGTTTTGAAGCAATTGTTCCACAGAACATATTTCCATGTTAGAGTGATTCAGGATGTTGCTGGTGCTTCCATAGCCGGAGCATTG
AAAAATGTTGTTGCTATTGCTGCTGGTTTCGTCGAGGGTGCCGGCTGGGGAGACAATGCCAAGGCTGCGGTGATGAGAATAGGACTCAAGGAG
ATTATTCACTTTGCTTCTTACTACAAGAAGTTTGGAATCAAGGCTCTTGTGGACCCTCAATCCACCACATTTACCGAAGAAAGTGCCGGTGTTGC
CGACTTGATCACCACCTGTTCCGGTGGTAGAAACGTCAAGGTTGCCCGTTACATGATCGAGCACAAGGTGGATGCTTGGGAAGCCGAAAAGAC
TTTGCTCAATGGTCAATCATCGCAAGGTATTTTGACCGCAAAAGAGGTGCACGAATTGTTGGAAAACTACGACTTGAAACAAGAGTTTCCTTTG
TTCGAGGCCACATACAAGGTGATCTACGAGAACACCGATGTCAACGACTTTCCAACGGTGTTGGAAGCCGACGATTAA

D_103937 ATGGTTTTGCCTCACGAGCCCGAATTTCAACAAGCTTACAACGAATTGGTTTCCGCTTTGGAGGAATCCACCTTGTTCCAAGAACACCCTCACT
ACAAGAAGGTGATTCCTGTTGTTTCAGTTCCCGAAAGAATTATTCAATTCAGAGTTTCATGGGAAAATGACAAGGGAGAAATCGAGGTGAACA
ATGGTTTCAGAGTCCAATTCAACTCGGCTCTTGGTCCTTACAAGGGAGGATTGAGATTCCACCCAACCGTCAACTTGTCGGTGTTGAAATTTTT
GGGATTTGAACAAATTTTCAAAAATGCCTTGACCGGTCTCTCCATGGGTGGAGGTAAAGGTGGATGTGACTTCAACCCCAAGGGAAGAAGTGA
CGCTGAAATTAGAAGATTCTGTGTTGCTTTCATGAGACAATTGGCCAGATACATTGGTGCCGACAGAGATGTTCCTGCTGGAGACATTGGTGTTG
GTGGTCGTGAAGTTGGTTACTTGTTCGGTGCCTACAAGCAAATGCAAAACAACTGGTCCGGTGTTTTGACCGGTAAGGGTTTGAGCTGGGGTG
GTTCTTTGATCCGTCCTGAAGCCACCGGTTACGGTACTGTCTACTATGTGGAGAAGATGATTGAAAAGGCCACTGGTGGAAAGGAGACTTTCAA

GGGTAAGCGTGTTGCCATTTCTGGTTCTGGTAACGTTGCCCAATACGCTGCTTTGAAGGTTATCGAATTGGGAGGAACTGTGGTTTCTTTGTCTG
ATTCTAAGGGAGCTCTCATTTCACAATCTGGTATTGTGCCTGAGCAGGTTGAGGCCATTGCTGCCGCTAAGCTCAAGTTCAAGTCATTGGAAGA
AATCTGCAAAGAATCTGCTTCCATTTTCTCGGGCAAAACCGAGTACATTGCCGGTGTCCGTCCATGGACCAAGGTTGGTCAAGTTGACGTAGCA
TTGCCATGTGCCACCCAAAACGAAGTCAGCGGTGACGAAGCTAAGGCTTTGGTTGACGCTGGATGCAAGTACATTGCTGAAGGTTCCAATATG
GGATCCACCGTCGAGGCTATCGACGTCTTCGAGGCTAACAGATCCAAGAATGTTTGGTATGCTCCAGGTAAGGCCGCCAACTGTGGTGGTGTTG
CCGTTTCTGGTTTGGAAATGGCCCAAAACTCTCAAAGAGTTTCCTGGACTTCTGAACAAGTTGACGAAAAGTTAAAGAACATCATGTACACCT
GTTTCGAGAACTGCTACAACACTGCTATCAAGTACTCTACCGAGAAGAGTGCTTCTGGCTTGCCATCTTTGTTGCAGGGTGCTAACATTGCTGGT
TTCATCAAGGTTGCCGATGCCATGTTTGACCAGGGTGAGGTGTTTTAG

D_103507 ATGTCCGAAATAAACTTCACTCCACTTAAAAACACCAAGGTTTTCCAGCCGATTCAAGTTGGAAAAAACCTCCTTTCCAACCGAATTTTTATGC
CCCCTCCACAAGAACCAGAGCATTGGACGATCGGACTCCTTCGAACTTGCAATTGCGCAACTACGACGAAAGAACAAAGTATGCTGGTTCGCT
TGTGGTCACCGAAGCCACTTTTTCATTTCCTCAGGCCGGAACCATGGCAGGAGTTCCGGGAATATATACCCCAGAACACACCAAAGGATGGAA
AAAAATTGTGGATAAGGTTCACGAGAACAATTCGTTTATTGCAATTCAGCTTTGGAACTTGGGTCGACTTGATAACCCCAAAGATTTGAAGGCA
GTAGGCTTACCCTACTTGGCACCTTCAGCCATTTATCCCGACAAAGATGCTCGGGAAGAAGCCGAGGCTGCGAATAATCCTATTAGAGCATTGAC
CGAAGAGGAAATCCACAACCAGATTTATGTGGAATACACCACGGCTGCAAAAAATGCCGTTGAGGCTGGGTTCGACTACTTGGAGTTACATGG
TGCTCACGGCTACTTGTTGCACCAATTCTTGGAAGATACCTCCAACCAAAGGACAGACAAGTATGGTGGATCGGTAGAGAACAGAGCCAGGTT
TGTGTTGGAGCTCATTGACCATCTTATTCCTATAGTTGGTGCTGACAAACTTGCTATTCGTCTTTCTCCATGGGTGACTATTAAAGGTATGCCTGGT
ATTCATGGTGATACCCATCCATTGACCACCTACAGCTACTTGCTACACGAGCTTGAAAAACGGGCTAAAGCCGGGAATCGGTTAGCCTACATTTC
CATTGTAGAGCCTAGAGTCAATGGGTCTACTACTCTTGAAACCAAAGACCAAACTGGAGACAATGGTTTTGTTGAAGATATTTGGAAGGGAACC
ATTCTCAAGGCGGGAAACTACACATATGATGCTCCAAAGTTCAATCTGGTGATTAAAGACGTTGAAAACGACCGTACGTTAGTCGGGTTCAGTC
GCTATTACGTCTCGAATCCAGACTTGGTTCAGCGATTGAAGGACGGAAATCCACTCAAGCCTTACGACCGTTCTCTCTTTACCGAAAGGACGA
CTGGGGGTACAATACTTATCCATACGAGGGACAAACAGAAGAGGAAATAGAGGCTGCAAAAAACAGAAAGCCAAAGCCTATTGGGGCCAAGG
CATAA

D_103508 ATGCTGGCAATTAAAGTTAAACCCCTCGGGGACACAAAGGTGTTTGAGCCTATACAGGTGGGAAAGAATACTCTTTCCAATAGACTTTTCATGT
GCCCAACTACAAGACTCAAGGCTTTAGAAGACGGTACTCCATCGAATTTGGCATTGCAACTCTATGACGAAAGAAGCAAGTTTCCCGGCTCGCT
CGTGACTACTGAAGGAACTTTCACTTATGAAGAGGGTCAAGTATGGGAAAGAACTCCAGGAATTTATACTGAAAGACATATTGAAGCATGGAAA
AAAATTGTCGATAAAGTTCATGAAAACAAATCTTTCATTTCACTCCAGTTGTTCAATTCGGGCCGTGTAGCCGACCCAACCATTGCAGACAACA
AGAATCATCCATTTGTTGCTCCATCGGCTATTTACCATGATGAGGAAACGAAAAAAGCTGCCATTGCTGCTGGCAATCCTTTGCGAGAATTAACC
TTGGATGAAATCCACGATATTATTAACAACAAGTACCCCAAAGCTGCCCATAATGCTCTTAGAGCCGGGTTCGACTATGTCGAAGTCCACGCTGC
AAACGGCTACCTTCCCAACCAATTTATTGACGTTGCCAGTAATCAGCGCACAGACCAATATGGCGGTTCGATTGAAAATAGAGCCAGGTTTGTG
TTGGAAATCATTGACAAATTGACTGCTGAGATTGGTGCCGACAAGATTGGGCTCAGGATTTCTCCATGGTCGACATTCCAGGGCATGCTGACCA
AAGGTGCAGAGATCGATCCATTGACCACTTACAGTTACATTTTACACGAATTGGAAAAAAGAGCTCAGAAGGGAAATAGGTTGGCGTATGTGTC
CATTATCGAGCCTAGAGTTGATGGAAACTCCACTGTCAAGAAAGAACACCAAGTTGGTGACAATTCGTTTGTCTACGATATTTGGAAGGGCACA
GTTTTGAGAGCGGGTGGATACACTTATGATGCCCCAGAATTCAAACGAGTTGAAACCGATCTTGCCAACGATAGAACAGTCATTGGTTTCTGTC
GTTACTATATCTCCAATCCAGATTTGGTTGAAAGGCTTAAAAATGGCTGGGATCTCCAACCATACGACAGGCCCTCTTTTTATAGAAGCGACGATT
ACCGCTACAATACCTATTCGTTCCATGGAGAGCCCCCTAGAGACGAGGAATCCGGCAAGAATAGAAAGCCTGAGCCAATTGCTGCTTAA

D_103084 ATGTCTGACGCTTCTGGCGAGCTACACAGAAGAAGGACTGCTAGTGGCTCACTCTATGGATCGGAAATAGATAGTTTCCATTCCGTAACTCAAA
ACTCAAAATGGAAGAATCTCAGACAAAAATCATTCACAATACTTAGAAAGTATGGGTCGTTTATAGGACCTGGTATCATGGTTTCAGTGGCATAT
ATGGACCCAGGTAACTATGCCACAGGTATAACAGCTGGTGCCTCCAACAGATTCTCCCTTTTATTCATCGTTCTAATTTCCAATATCATTGCCATTT

TTCTTCAAAGTTTATGCATCAAGTTGGGCTCAGTAACCGGCTACGATCTTGCCCGTTGTTGTCGAGAATACCTTCCCAAGTGGCTCAATATCATTC
TCTGGATCCTTGCGGAAAGTGCAATCATTGCAACTGATGTGGCTGAAGTTATAGGTTCAGCTATTGCCCTTAATATTCTTCTTAAAATTCCACTTC
CAGCAGGCGTGGTAATTACAATAGTGGACGTTTTATTTGTGCTAATGGCGTATCGCAACGACACTTCCTCTACCAAGTTTGTCAAGATGTTTGAG
TATGCAGTTGCTTGTCTTGTGGTGGCCGTGGTGGTTTGCTTCGCAGTGGAAATTGCCCATTTACCAGTCACTTCAGAAATGGTTCGTGAGATCTT
CAGAGGTTATGCTCCTTCGAAAGAAATGGTCCAGGGAAGTGGCTTGACCATTGCTACTGGTATCATTGGATCCACAGTTATGGTACACTCATTAT
TTTTGGGTTCTGGTCTCGTTCAGCCTAGATTGAGAGAGTACGATGTAACCCACGGTCTTGTGGATCTTGATCTGGTATGTTCTGAGGACGAAACT
ACAGCAGATACTGTCTCAGAAAAATCGTCTACCAGAGTTAAAACTACTCGTATCGACAAAGAAGCTCTCTTCTTTTACAAGAGTTACAAACCAT
CGTACCAATCAATTCAATACTCGTTGAAATACTCAATTATTGAACTTGTGGTTACGCTTTTCACATTTGCACTTTTCGTCAACTCGGCTATCTTGGT
GGTGGCTGGAGTTACCCTCAACGATACCCCAGAAGCCATCGATGCCGACTTGTACACTATTCATGCATTGCTCTCCAAAAATTTGGCACCTGTTG
TAGGCACAGTTTTCATGCTTGCGTTGCTTTTCAGTGGTCAAAGTGCTGGTATCGTCTGTACTATTGCTGGACAAATGGTAAGTGAGGGCCATATC
AATTGGAAGCTTAAACCATGGGTGAGAAGACTAGTGACAAGAGCCATCTCAATTATACCTTGTTTGGCCATTTCTGTTGGTATTGGACGGTCCGG
TTTGGGACTCGCCTTGAACGTTTCACAGGTTGTGATTTCCATTCTTCTTCCCCCATTAACGGCTCCTCTTATCTACTTTACATGCAAAAAGTCCAT
CATGAAAGTGCCATTACCCAAGGAGATGCAGTCAGATGATGAAGACGAAGTAAAATACAAATATCTACACAACAATTGGCTCACATCCATCATT
GTCTTTGCCATCTGGCTCTTTGTGCTGGCATTGAATATCTACGCGATTGTTGACATGGCCAAAAATGGTGTGGCCGGCAGTTAG

Table S3 DEGs related to metabolism of *M. guilliermondii* A4 in "S1 vs S0" and "S2 vs S0" groups.

Description	Gene ID	Annotation (Protein ID)	log_2 FC	
			S1 vs S0	S2 vs S0
Sugar transportation and utilization	D_104547	Hexose transporter 2 (P53387)	2.206	1.553
	D_102607	High-affinity glucose transporter 1 (A0A1D8PCL1)	1.193	——
	D_102333	Probable glucose transporter rco-3 (Q92253)	1.165	——
	D_105325	Sugar transporter STL1 (P39932)	-1.679	——
	D_104687	Low-affinity glucose transporter (P18631)	-2.410	——
	D_104584	Sugar transporter STL1 (P39932)	——	1.218
	D_104023	Sugar transporter STL1 (P39932)	——	-1.006
	D_105427	Probable sucrose utilization protein SUC1 (P33181)	——	-1.135
	D_100896	Glucose transporter GlcP (A0A0H2VG78)	——	-1.313
	D_102664	Sugar transport protein 12 (O65413)	——	-1.321
	D_102555	Probable sucrose utilization protein SUC1 (P33181)	——	-1.325
	D_104356	Probable sucrose utilization protein SUC1 (P33181)	——	-1.603
	D_103907	Sugar transporter STL1 (P39932)	——	-1.811
	D_103142	High-affinity glucose transporter HXT2 (P23585)	——	-1.820
	D_102377	Hexose transporter HXT8 (P40886)	——	-1.951
	D_103976	Sugar transporter STL1 (P39932)	——	-2.750
	D_102554	Maltose permease MAL31 (P38156)	——	-1.775
	D_105520	Maltose permease MAL31 (P38156)	——	-1.961
	D_101515	Maltose permease MAL61 (P15685)	——	-2.389
	D_100515	Maltose permease MAL61 (P15685)	-1.557	-3.716
	D_100514	Maltose permease MAL61 (P15685)	-2.118	-4.209
	D_100516	Maltose permease MAL61 (P15685)	-2.193	-6.957
Cytochrome P450	D_101278	Cytochrome P450 61 (P54781)	1.257	1.447
	D_105475	Cytochrome P450 52A12 (Q9Y757)	-1.076	——
	D_105324	Cytochrome P450 52A12 (Q9Y757)	-1.470	-1.555
	D_100696	Cytochrome P450 regulator dap1 (O13995)	——	-1.017
	D_105529	Cytochrome P450 52A12 (Q9Y757)	——	-1.052
	D_102610	Cytochrome P450 52A13 (Q9Y758)	——	-1.068
	D_102378	Cytochrome P450 52A12 (Q9Y757)	——	-1.592
Others	D_104882	NADH-ubiquinone oxidoreductase assembly factor N7BML (Q6C7L6)	——	1.393

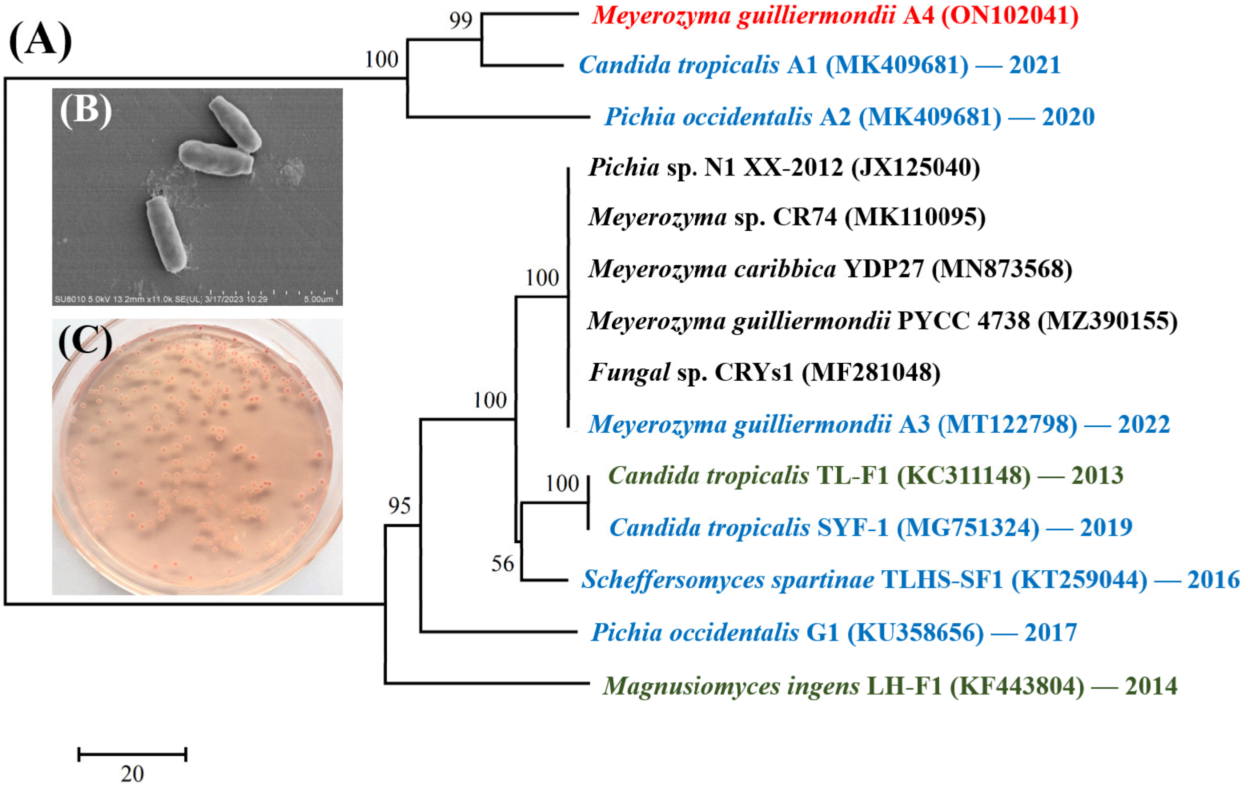

Figure S1 *M. guilliermondii* A4: (A) phylogenetic tree (Neighbor-Joining method); (B) scanning electron microscopic (SEM) micrograph of cells; (C) colonies on solid agar plate. The yeasts which were highlighted in blue and green color were the ones we have isolated, researched and reported (in the year followed by the corresponding GenBank accession numbers). Those in green color were non-halotolerant yeasts and those in blue color were halotolerant ones.

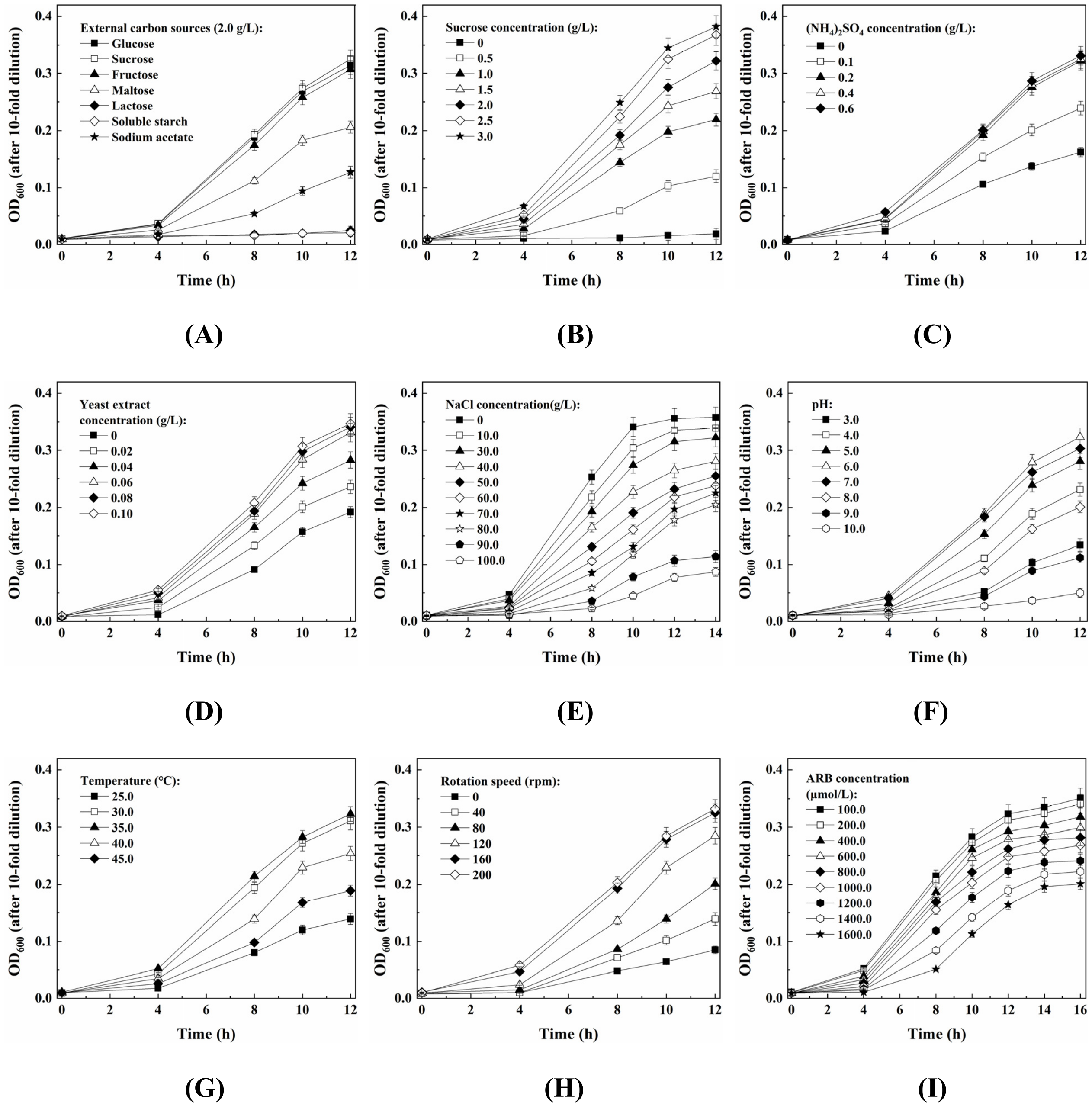

Figure S2 Optimization of the conditions for cell growth of *M. guilliermondii* A4: (A) type of external carbon source (2.0 g/L); (B) sucrose concentration; (C) $(NH_4)_2SO_4$ concentration; (D) yeast extract concentration; (E) NaCl concentration; (F) pH; (G) temperature; (H) rotation speed; (I) ARB concentration.

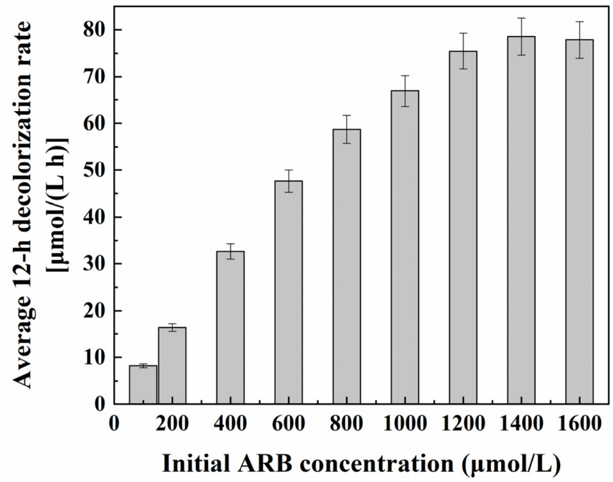

Figure S3 Average 12-h decolorization rate of ARB of different initial concentration.

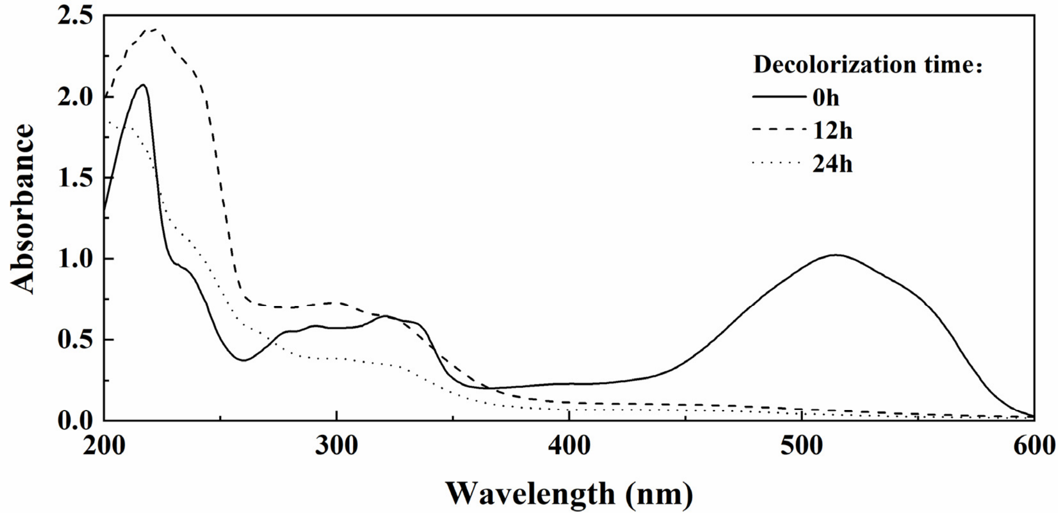
Absorbance
Wavelength (nm)
Decolorization time:
0h
12h
24h
2.5
2.0
1.5
1.0
0.5
0.0
200
300
400
500
600

(A)

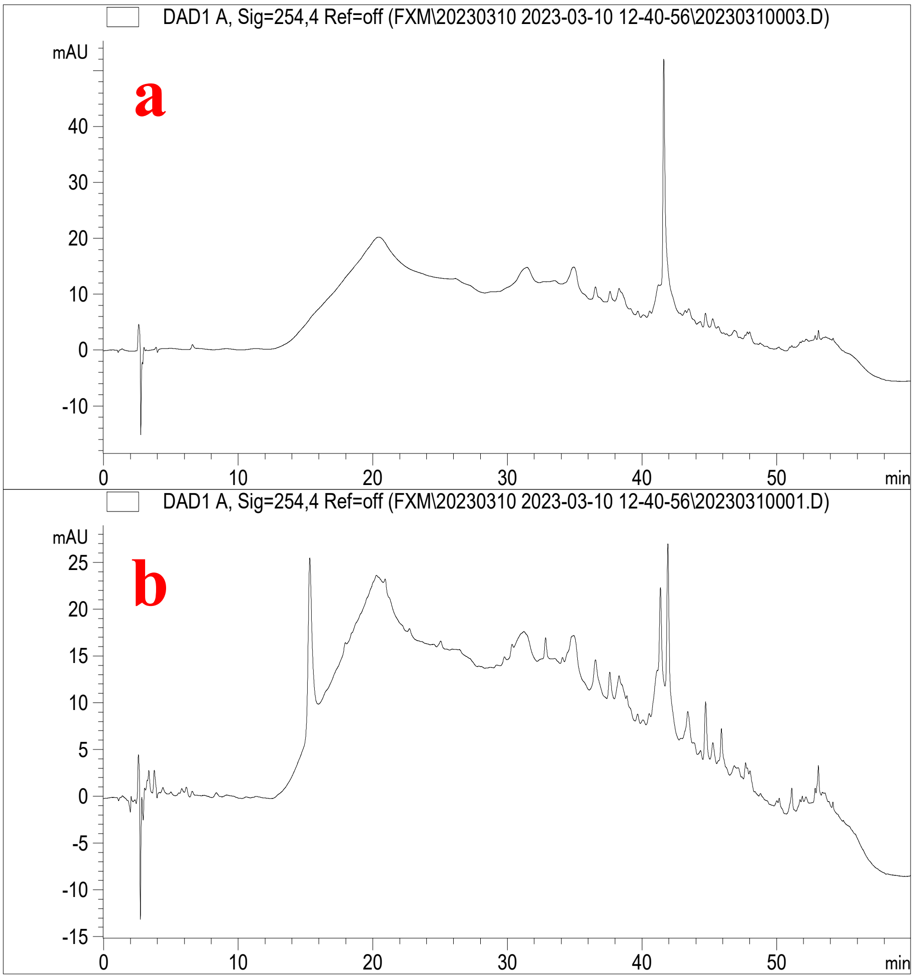
DAD1 A, Sig=254,4 Ref=off (FXM\20230310 2023-03-10 12-40-56\20230310003.D)
mAU
a
min
DAD1 A, Sig=254,4 Ref=off (FXM\20230310 2023-03-10 12-40-56\20230310001.D)
mAU
b
min

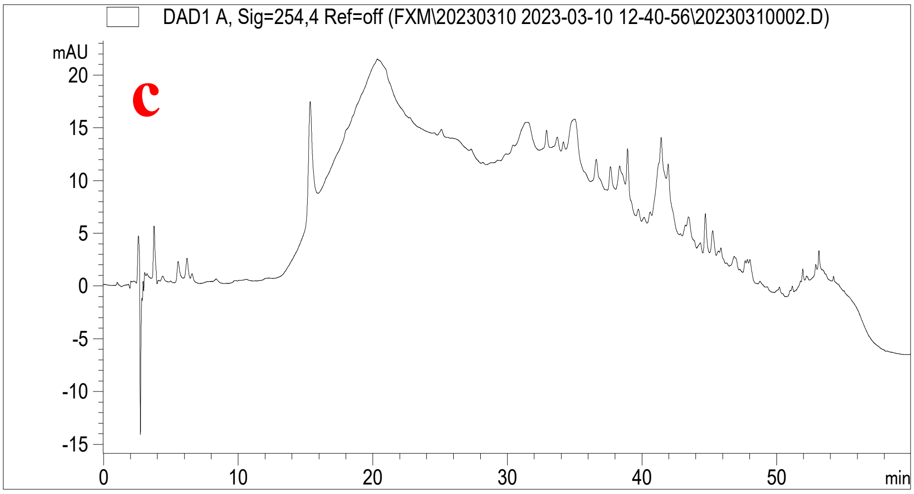
DAD1 A, Sig=254,4 Ref=off (FXM\20230310 2023-03-10 12-40-56\20230310002.D)
mAU
c
min

(B)

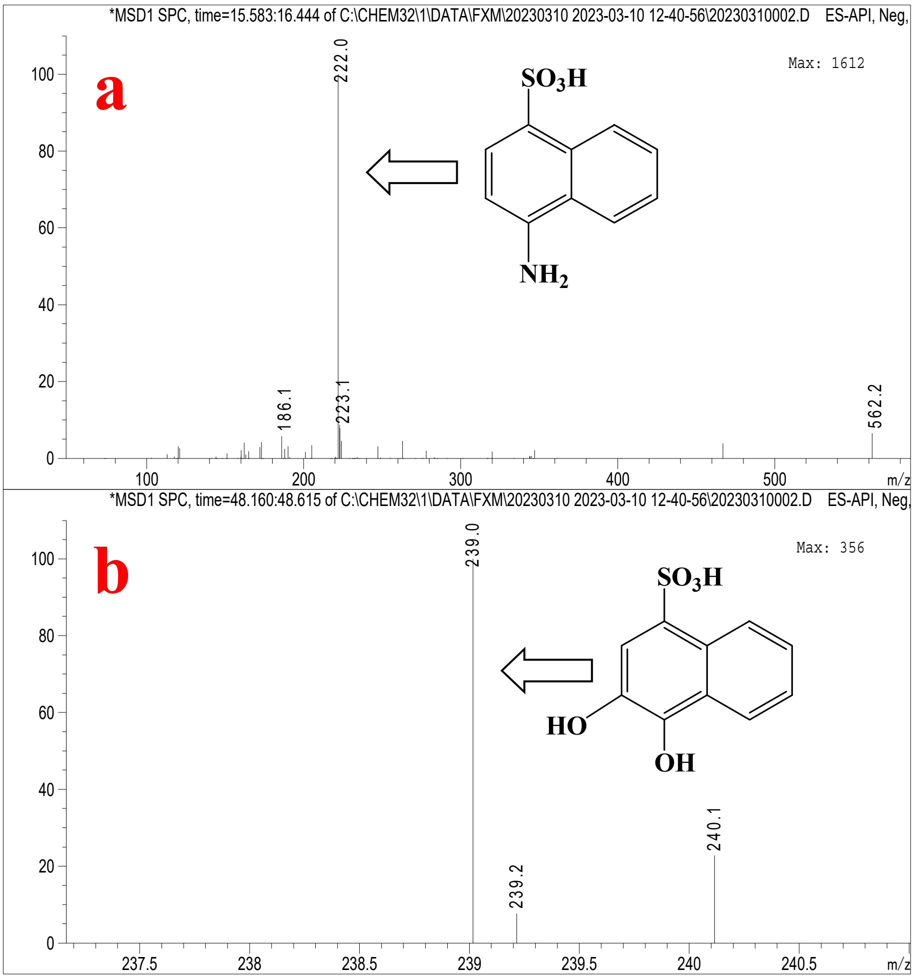
*MSD1 SPC, time=15.583:16.444 of C:\CHEM32\1\DATA\FXM\20230310 2023-03-10 12-40-56\20230310002.D ES-API, Neg,
Max: 1612
a
222.0
186.1
223.1
562.2
SO3H
NH2
m/z
*MSD1 SPC, time=48.160:48.615 of C:\CHEM32\1\DATA\FXM\20230310 2023-03-10 12-40-56\20230310002.D ES-API, Neg,
Max: 356
b
239.0
239.2
240.1
SO3H
HO
OH
m/z

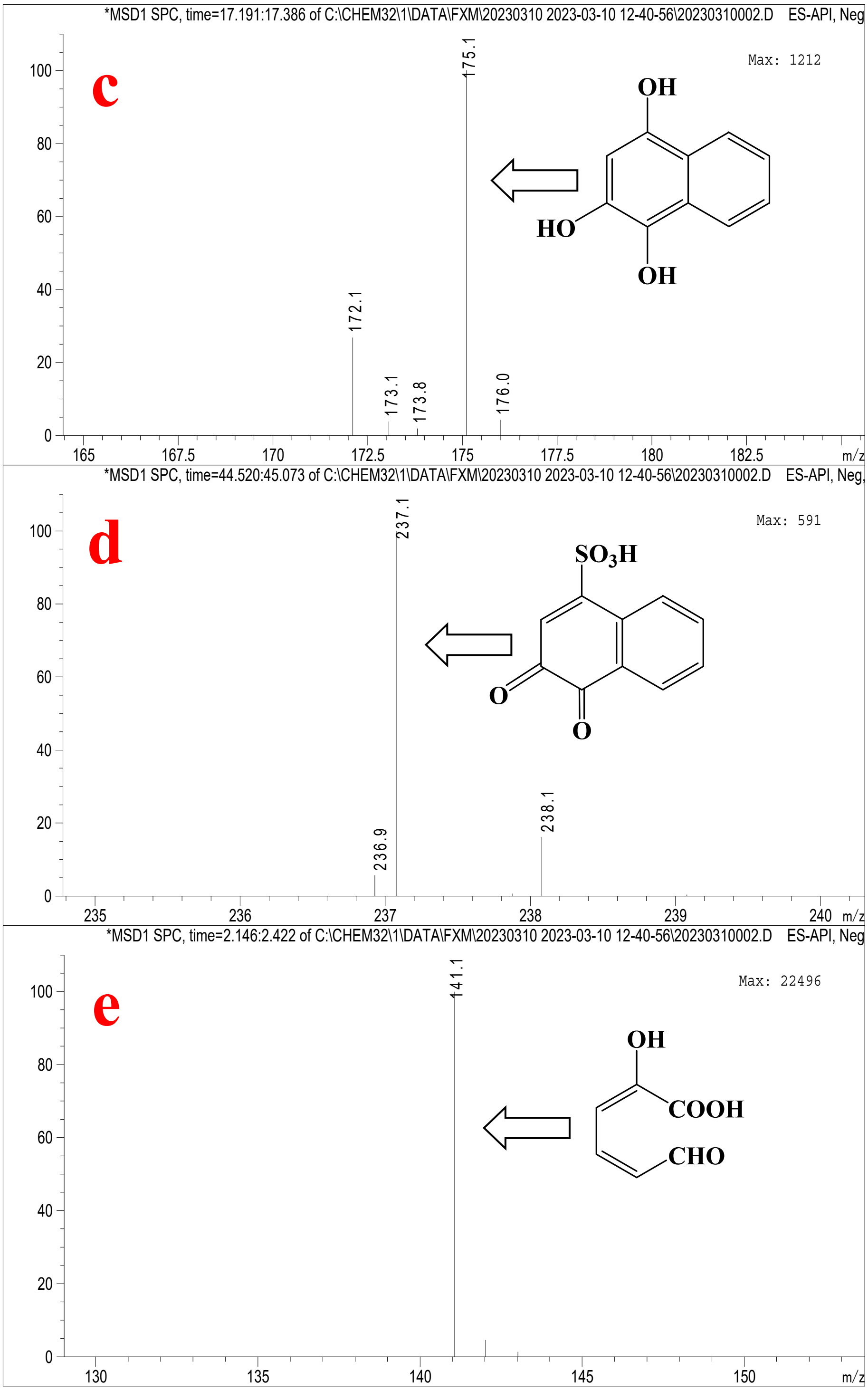
*MSD1 SPC, time=17.191:17.386 of C:\CHEM32\1\DATA\FXM\20230310 2023-03-10 12-40-56\20230310002.D ES-API, Neg
c
Max: 1212
175.1
172.1
173.1
173.8
176.0
OH
HO
OH
m/z
*MSD1 SPC, time=44.520:45.073 of C:\CHEM32\1\DATA\FXM\20230310 2023-03-10 12-40-56\20230310002.D ES-API, Neg,
d
Max: 591
237.1
236.9
238.1
SO_3H
O
O
m/z
*MSD1 SPC, time=2.146:2.422 of C:\CHEM32\1\DATA\FXM\20230310 2023-03-10 12-40-56\20230310002.D ES-API, Neg
e
Max: 22496
141.1
OH
COOH
CHO
m/z

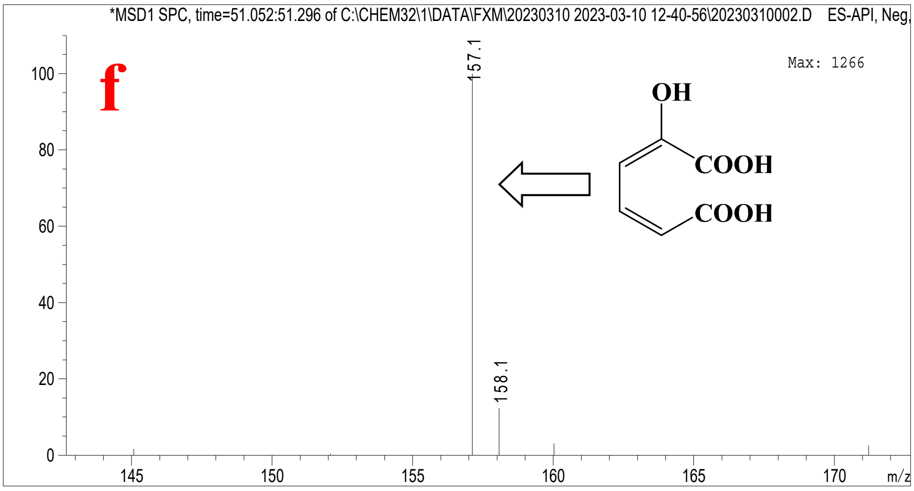

(C)

Figure S4 (A) UV-Vis scanning spectrum of ARB before and after decolorization for 12 h and 24 h by *M. guilliermondii* A4. (B) HPLC spectrums of ARB (a) before and after decolorization for (b) 12 h and (c) 24 h. (C) MS of six possible decolorization intermediates: (a) 4-aminonaphthalene-1-sulfonic acid; (b) 3,4-dihydroxynaphthalene-1-sulfonic acid; (c) naphthalene-1,2,4-triol; (d) 3,4-dioxo-3,4-dihydronaphthalene-1-sulfonic acid; (e) (2*E*,4*Z*)-2-hydroxy-6-oxohexa-2,4-dienoic acid; (f) (2*E*,4*Z*)-2-hydroxyhexa-2,4-dienedioic acid.

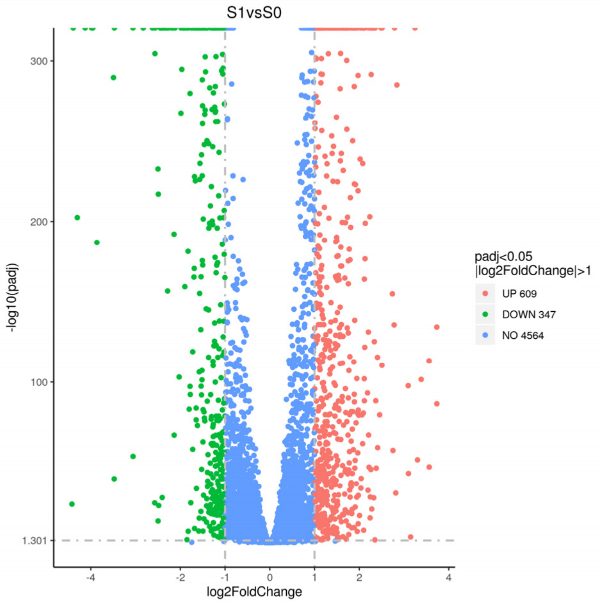

(A)

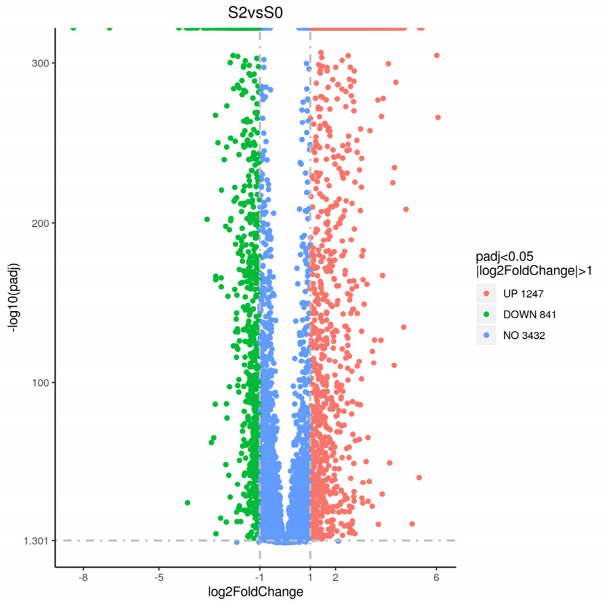

(B)

Figure S5 Volcano map of DEGs in comparisons of (A) S1 and S0, (B) S2 and S0**.**

Text S1. Isolation and identification of halotolerant azo-dye-degrading yeast

The pure yeast strain capable of decolorizing azo dye in brine was isolated from sea mud samples collected in Dalian, China (38.88 °N, 121.57 °E). Before yeast isolation, the sea mud microbial community was acclimated in 250 mL shaking flasks for continuous decolorization of different azo dyes (gradually increased from 40.0 μmol/L to 200.0 μmol/L) in the medium (100 mL) containing (g/L): sucrose 2.0, $(NH_4)_2SO_4$ 1.0, yeast extract 0.1, K_2HPO_4 1.0, $MgSO_4 \cdot 7H_2O$ 0.5 and NaCl 30.0. Other culture conditions were: temperature 35 °C, rotation speed 160 rpm, initial pH 6.0 and acclimatization time 36 d. Pure strains were isolated using spread-plate method on the agar (2.0%, w/v) medium plates containing (g/L): sucrose 2.0, $(NH_4)_2SO_4$ 1.0, yeast extract 0.1, K_2HPO_4 1.0, $MgSO_4 \cdot 7H_2O$ 0.5 and NaCl 30.0, as well as azo dye (μmol/L) 40.0. The strain with the highest decolorization efficiency was selected as the target yeast for further study. The target yeast strain was identified through 26S rDNA and Internal Transcribed Space (ITS) sequencing method which was performed by Sangon Biotech Co., Ltd. (Shanghai, China). Then a phylogenetic tree was constructed using Neighbor-joining (NJ) method by MEGA (Version 4.0) with 10,000 bootstrap replicates. Released 26S rDNA sequences of some yeast strains in GenBank database (https://www.ncbi.nlm.nih.gov/genbank/) which exhibited > 99% homology to the isolate according to the BLAST results and those of the yeasts which were have yet isolated and reported by us were chosen for constructing the phylogenetic tree. In addition, morphology observation of the yeast cells was performed with a field emission scanning electron microscope (Hitachi SU8010, Japan).

Text S2. Transcriptomics analysis

S2.1. RNA isolation, cDNA library preparation and transcriptome sequencing

RNA integrity was assessed using the RNA Nano 6000 Assay Kit of the Bioanalyzer 2100 system (Agilent Technologies, CA, USA). Total RNA was used as input material for the RNA sample preparations. Briefly, mRNA was purified from total RNA using poly-T oligo-attached magnetic beads. Fragmentation was carried out using divalent cations under elevated temperature in First Strand Synthesis Reaction Buffer (5X). First strand cDNA was synthesized using random hexamer primer and M-MuLV Reverse Transcriptase (RNase H-). Second strand cDNA synthesis was subsequently performed using DNA Polymerase I and RNase H. Remaining overhangs were converted into blunt ends via exonuclease/polymerase activities. After adenylation of 3' ends of DNA fragments, Adaptor with hairpin loop structure were ligated to prepare for hybridization. In order to select cDNA fragments of preferentially 370-420 bp in length, the library fragments were purified with AMPure XP system (Beckman Coulter, Beverly, USA). Then PCR was performed with Phusion High-Fidelity DNA polymerase, Universal PCR primers and Index (X) Primer. At last, PCR products were purified (AMPure XP system) and library quality was assessed on the Agilent Bioanalyzer 2100 system.

The clustering of the index-coded samples was performed on a cBot Cluster Generation System using TruSeq PE Cluster Kit v3-cBot-HS (Illumina Inc., USA) according to the manufacturer's instructions. After cluster generation, the library preparations were sequenced on an Illumina Novaseq platform and 150 bp paired-end reads were generated.

S2.2. Quality control and mapping of transcriptome sequencing results

Raw data (raw reads) in the ".fastq" format were firstly processed through FASTQ software. In this step, clean data (clean reads) were obtained by removing reads containing adapter, reads containing ploy-N and low-quality reads from raw data. At the same time, Q20, Q30 and GC content the clean data were calculated. All the downstream analyses were based on the clean data with high quality.

The draft genome sequence of *Meyerozyma guilliermondii* A3 (with the accession ID of JAKFAP000000000) was referenced for mapping. Reference genome and gene model annotation files were downloaded from genome website directly. Index of the reference genome was built and paired-end clean reads were aligned to the reference genome using Hisat2 v2.0.5, because that this mapping tool can generate a database of splice junctions based on the gene model annotation file and thus can provide better mapping results than other non-splice mapping tools. The mapped reads of each sample were assembled by StringTie (v1.3.3b) (Pertea et al., 2015) in a reference-based approach. StringTie uses a novel network flow algorithm as well as an optional de novo assembly step to assemble and quantitate full-length transcripts representing multiple splice variants for each gene locus. FeatureCounts v1.5.0-p3 was used to count the reads numbers mapped to each gene. And then FPKM of each gene was calculated based on the length of the gene and reads count mapped to this gene. FPKM, expected number of Fragments Per Kilobase of transcript sequence per Millions base pairs sequenced, considers the effect of sequencing depth and gene length for the reads count at the same time, and is currently the most commonly used method for estimating gene expression levels.

S2.3. Analysis of differentially expressed genes (DEGs) and metabolic pathways

Differential expression analysis of two conditions/groups (two biological replicates per condition) was performed using the DESeq2 R package (1.20.0). DESeq2 provide statistical routines for determining differential expression in digital gene expression data using a model based on the negative binomial distribution. The resulting *P*-values were adjusted using the approach by Benjamini and Hochberg (1995) for controlling the false discovery rate. Genes with an adjusted *P*-value ≤ 0.05 found by DESeq2 were assigned as differentially expressed. DEGs were identified as those with an FDR of <0.001 and a RPKM (reads per kilobase of exon model per million mapped reads) ratio of the two samples of > 2. The fold change of DEGs was shown as the $\log_2$ Fold Change ($\log_2$ FC) of gene abundance via comparison of the SMF-stimulated sample and the control, and the screening criterion was $\log_2$ FC ≥ 1.

Gene Ontology (GO) enrichment analysis of differentially expressed genes was implemented by the clusterProfiler R package, in which gene length bias was corrected. GO terms with corrected *P*-value < 0.05 were considered significantly enriched by differential expressed genes. KEGG is a database resource for understanding high-level functions and utilities of the biological system, such as the cell, the organism and the ecosystem, from molecular-level information, especially large-scale molecular datasets generated by genome sequencing and other high-through put experimental technologies (http://www.genome.jp/kegg/). We used clusterProfiler R package to test the statistical enrichment of differential expression genes in KEGG pathways.

S2.4. Quantitative Real-Time PCR (QRT-PCR) validation

In order to confirm the reliability of DEGs identified by RNA-Seq, genes with significant changes and related functions were validated through QRT-PCR by Sangon Biotech Co., Ltd., (Shanghai, China) according to the method described by Yong et al. (2013). Primers for QRT-PCR were designed according to their gene sequences according to the transcriptome sequencing results. QRT-PCR was performed using a LightCycler 480 instrument (Roche Diagnostics, Mannheim, Germany). The reaction measurements were performed in biological triplicate. The results were displayed relative to the expression levels of reference gene (18S rRNA) in each sample using the $2^{-\Delta\Delta Ct}$ method according to the method described by Schmittgen and Livak (2008).

References

Benjamini, Y., Hochberg, Y., 1995. Controlling the false discovery rate: a practical and powerful approach to multiple testing. J. Roy. Statist. Soc. Ser. B 57, 289–300.

Pertea, M., Pertea, G.M., Antonescu, C.M., Chang, T.C., Mendell, J.T., Salzberg, S.L., 2015. StringTie enables improved reconstruction of a transcriptome from RNA-seq reads. Nat Biotechnol. 33, 290–295. https://doi.org/10.1038/nbt.3122.

Schmittgen, T.D., Livak, K.J., 2008. Analyzing real-time PCR data by the comparative CT method. Nat. Protoc. 3, 1101–1108. https://doi.org/10.1038/nprot.2008.73.

Yong, X., Zhang, R., Zhang, N., Chen, Y., Huang, X., Zhao, J., Shen, Q., 2013. Development of a specific real-time PCR assay targeting the poly-γ-glutamic acid synthesis gene, pgsB, for the quantification of *Bacillus amyloliquefaciens* in solid-state fermentation. Bioresour. Technol. 129, 477–484. https://doi.org/10.1016/j.biortech.2012.11.092.
